# Short‐Term Severe Energy Restriction Promotes Molecular Health and Reverses Aging Signatures in Adults With Prediabetes in the PREVIEW Study

**DOI:** 10.1111/acel.70123

**Published:** 2025-06-16

**Authors:** Maria Lastra Cagigas, Andrius Masedunskas, Yao Lin, Samantha J. Emery‐Corbin, Jumana M. Yousef, Laura F. Dagley, Sam Olechnowicz, Rory Bowden, Rachael Hayward, Gary Low, Roslyn Muirhead, Jennie Brand‐Miller, Mikael Fogeltholm, Anne Raben, Marco Demaria, Stephen J. Fuller, Luigi Fontana

**Affiliations:** ^1^ Charles Perkins Centre The University of Sydney Sydney Australia; ^2^ Sydney Medical School Nepean, Faculty of Medicine and Health The University of Sydney Sydney Australia; ^3^ European Research Institute for the Biology of Ageing (ERIBA), University Medical Center Groningen (UMCG) University of Groningen (RUG) Groningen the Netherlands; ^4^ Advanced Technology and Biology Division The Walter and Eliza Hall Institute of Medical Research Melbourne Victoria Australia; ^5^ Department of Medical Biology University of Melbourne Melbourne Victoria Australia; ^6^ School of Life and Environmental Sciences and Charles Perkins Centre The University of Sydney Sydney New South Wales Australia; ^7^ Department of Food and Nutrition University of Helsinki Helsinki Finland; ^8^ Department of Nutrition, Exercise and Sports, Faculty of Science University of Copenhagen Frederiksberg Denmark

## Abstract

Prediabetes, characterized by impaired fasting glucose and/or glucose tolerance, is associated with organ damage, increased mortality, and accelerated aging, even before diabetes onset. Severe short‐term energy restriction while maintaining essential nutrient intake is among the most effective strategies for weight loss, metabolic health improvement, and delaying type 2 diabetes progression. Extracellular vesicles contribute to these metabolic benefits; however, the impact of energy‐restriction‐induced weight loss on the extracellular vesicle proteome remains incompletely understood. This study employed targeted and untargeted proteomics to investigate the effect of an 8‐week severely energy‐restricted diet on the plasma proteome in adults with prediabetes from Sydney, Australia, as part of the PREVIEW study. Circulating extracellular vesicles were enriched in plasma using an immunoaffinity‐based protocol. A total of 44 participants who achieved at least a 12% weight loss and provided informed consent were included in the study. Paired changes in over 2000 proteins between baseline and week 8 were analyzed. Following the intervention, multiple proteins associated with inflammation and senescence were significantly reduced, reversing the increase commonly associated with aging. The decline in inflammatory and senescence markers may have been mediated by extracellular vesicles, as indicated by significantly lower circulating levels of several vesicular markers. Additionally, several markers of protein synthesis downstream of mTORC1 and protein degradation were significantly reduced in vesicle‐enriched plasma, suggesting decreased intercellular secretion and/or trafficking. Overall, this study identifies a diet‐induced proteomic signature suggestive of reduced inflammation, lower senescence, and enhanced vesicle‐associated proteostasis, potentially conferring health benefits beyond glycemic control.

## Graphical abstract

Severe short‐term energy restriction in adults with prediabetes led to a significant reduction in circulating proteins linked to inflammation, senescence, and protein turnover. These changes, partly mediated by extracellular vesicles, suggest systemic dietary‐induced benefits beyond glycemic control, including improved aging biomarkers.

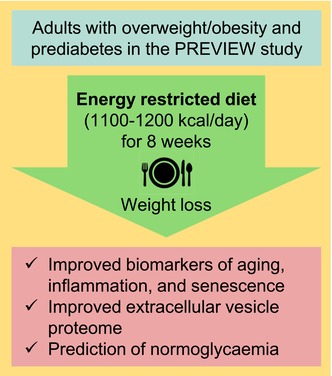

The global incidence of type 2 diabetes (T2D) continues to rise despite the disease's preventability through lifestyle changes (Goldman et al. 2024; Lean et al. 2018; McMacken and Shah 2017; Nakhleh et al. 2024; Raben et al. 2021; Sarwar et al. 2010; Silva et al. 2024; Sluijs et al. 2010; Thompson et al. 2024). Prediabetes, a metabolic condition marked by hyperglycemia or impaired glucose tolerance, causes early damage to organs, including the kidneys, eyes, heart, and arteries, and is associated with accelerated brain aging (Dove et al. 2024), well before a diagnosis of T2D (Nakhleh et al. 2024; Sarwar et al. 2010; Tabák et al. 2012). Meta‐analyses show that prediabetes significantly increases the risk of all‐cause mortality, cardiovascular disease, stroke, nephropathy, cancer, and neurological disorders (Huang et al. 2016; Schlesinger et al. 2022; Cai et al. 2020), highlighting the urgent need for effective strategies to prevent or reverse this condition. Such interventions are crucial for reducing cumulative metabolic and molecular damage, as well as preventing the progression to T2D and associated chronic diseases. Tissue and cellular damage in prediabetes results from multiple mechanisms such as insulin resistance, hyperglycemia, nutrient‐sensing dysregulation, and chronic inflammation (Dove et al. 2024; Tabák et al. 2012). These factors drive protein glycation, impaired insulin signaling, metabolic and oxidative stress, endothelial dysfunction, and cellular senescence. Dysregulated insulin/IGF‐1 signaling also affects mTORC1 activity, disrupting proteostasis (Cochemé and Gil 2024; Panwar et al. 2023; Zoncu et al. 2011; Holzenberger et al. 2003; Junnila et al. 2013; Katic and Kahn 2005). Together, these processes promote organ damage and accelerated aging. Additionally, obesity, which often accompanies prediabetes, exacerbates immune senescence (Brunelli et al. 2021; Chaib et al. 2021; Li et al. 2021; Shirakawa et al. 2016) and accelerates epigenetic aging in metabolically active tissues like the liver and visceral fat (de Toro‐Martín et al. 2019).

Lifestyle interventions, particularly energy restriction with optimal nutrient intake, are highly effective in reversing prediabetes (Nakhleh et al. 2024; Galaviz et al. 2022; Das et al. 2023; Huffman et al. 2022; Kraus et al. 2019; Khong and Kimpton 2020; Kim et al. 2020; Merra et al. 2017; Fontana et al. 2016, 2008; Fontana 2006; Heilbronn et al. 2006). Large‐scale clinical trials, including CALERIE, DiRECT, and PREVIEW, have consistently demonstrated that energy restriction enhances cardiometabolic health, improves glucose tolerance and insulin sensitivity, and can prevent or even reverse T2D progression (Lean et al. 2018, 2019; Raben et al. 2021; Huffman et al. 2022; Kraus et al. 2019). For instance, the CALERIE‐2 trial showed that a modest 13% energy restriction over 2 years led to a supraphysiological improvement in lipid profile, blood pressure, insulin sensitivity, and inflammatory and oxidative stress markers (Huffman et al. 2022; Kraus et al. 2019; Heilbronn et al. 2006). The DiRECT trial found that a severely restricted diet (~840 kcal/day) followed by sustained weight loss enabled T2D remission (Lean et al. 2018, 2019). In the PREVIEW study, adults with overweight/obesity and prediabetes who followed an 8‐week low‐energy diet followed by a 3‐year healthy eating and behavior change intervention reduced their diabetes risk from 13.5% to 3.0% (Raben et al. 2021). The severe short‐term energy restriction (1100–1200 kcal/day) employed in this study, which contrasts with the more modest long‐term energy‐restriction strategies like those used in CALERIE, was highly effective in inducing rapid weight loss and improving metabolic health. Despite these benefits, the biological adaptations of severe short‐term energy restriction remain incompletely understood.

Recent advances in high‐throughput proteomics now allow for the simultaneous measurement of thousands of plasma proteins with high specificity and sensitivity, providing a unique and unbiased opportunity to investigate the comprehensive molecular responses to energy restriction. This approach overcomes the limitations of previous studies that focused on a limited selection of proteins. This study conducted a plasma proteomic analysis on a subset of participants with overweight/obesity and prediabetes from the PREVIEW study in Sydney, Australia. Participants (*n* = 44) were selected based on achieving at least a 12% weight loss following the low‐energy diet, exceeding the PREVIEW protocol's 8% requirement by four percentage points (Raben et al. 2021). This higher threshold was chosen to maximize the detection of proteomic changes. The intervention involved a continuous low‐energy diet for 8 weeks, representing an approximate 40%–50% reduction in energy intake. Untargeted mass spectrometry combined with a depletion‐enrichment protocol was used to capture both free and extracellular vesicle‐bound proteins in plasma. Proteins associated with circulating extracellular vesicles (cEVs) were of particular interest, given the extracellular vesicles' role in mediating glycemic control in mice (Miotto et al. 2024). In addition, the Olink inflammation panel was used to systematically assess inflammatory proteins not captured by mass spectrometry, including senescence‐associated secretory phenotype (SASP) factors and aging‐related proteins. We hypothesized that energy restriction would induce a plasma proteomic shift characterized by decreased inflammation and senescence and maintenance of proteostasis.

## 1 Results

### 1.1 Participants: Adults With Overweight/Obesity and Prediabetes in Sydney, Australia

**FIGURE 1:**
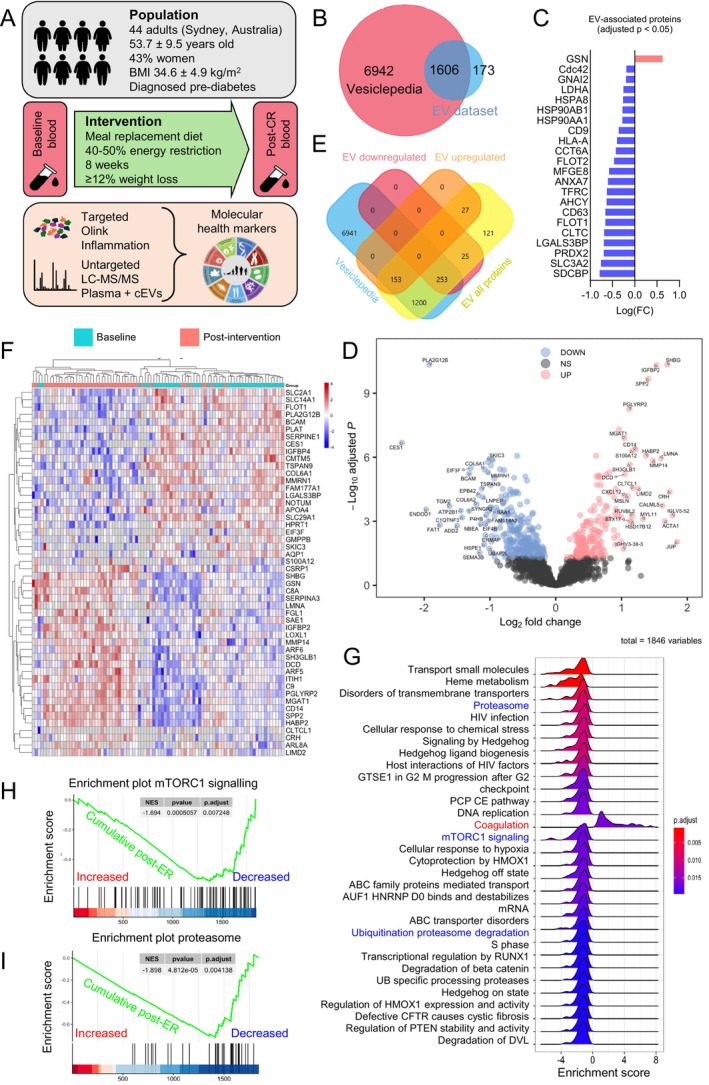
Energy‐restriction‐induced weight loss reduces circulating extracellular vesicle (cEV) markers. (A) Diagram of study design, population, intervention, and methodology. BMI, Body Mass Index; ER, Energy Restriction; cEV, circulating extracellular vesicles; LC–MS/MS, liquid chromatography with tandem mass spectrometry. (B) Overlap between cEV‐enriched plasma proteins from LC–MS/MS (EV dataset, blue) and proteins annotated in Vesiclepedia (pink). Vesiclepedia was accessed in October 2024. (C) All differentially expressed proteins (adj *p* < 0.05) from the EV dataset that belong to the “top 100 EV‐associated proteins” in Vesiclepedia. Significantly decreased (*n* = 21, blue) and significantly increased (*n* = 1, pink) proteins. (D) Volcano plot of cEV proteins from LC–MS/MS at week 8 relative to baseline, including significantly decreased (blue), unchanged (gray), and significantly increased (pink) (adj *p* < 0.05). Proteins with absolute FC ≥ 2 are named. (E) Overlap of differentially expressed cEV proteins from “D” with the Vesiclepedia database. (F) Top 50 significantly differentially expressed cEV proteins by adj *p*‐value, paired. Each row represents a significantly decreased (blue) or increased (red) protein. Each column represents a study participant at baseline (teal) and at week 8 (pink). (G) GSEA of significantly enriched pathways post‐intervention relative to baseline, including downregulated (negative enrichment score) and upregulated (positive enrichment score) pathways from the cEVs dataset. Adj *p*‐values are color‐coded. (H, I) Related to “G,” enrichment plots of the mTORC1 signaling pathway and the proteasome post‐CR. The green lines represent the cumulative enrichment score, with proteins increased on the left (red) and decreased on the right (blue). Adj *p* = 0.0072 and 0.0041, respectively. Input = cEV proteins.

In the Australian sub‐cohort of the PREVIEW trial, 595 participants were screened, of whom 195 met the eligibility criteria (men and women with overweight/obesity and prediabetes). Among these, 167 participants were enrolled and commenced the low‐energy diet intervention, with 151 completing the weight loss program. During this phase, participants adhered to a low‐energy diet (LED) based on the Cambridge Weight Plan, under the guidance of a dietitian. This diet, providing approximately 1110–1210 kcal per day (representing approximately 40%–50% energy restriction), resulted in a significant mean weight loss of 11.0 ± 3.5 kg, or 10.9% ± 2.6% of body weight (Table S1), as previously reported (Raben et al. 2021). Notably, 30% of participants achieved a weight reduction exceeding 12% and were selected for proteomic analysis to maximize the detection of protein changes associated with weight loss (Table 1 and Figure 1A). This subgroup (*n* = 44) exhibited demographic and clinical characteristics similar to the broader cohort, albeit with a different sex distribution (69.5% female in the whole cohort versus 43.2% female in the subgroup) and more pronounced weight loss. The subgroup had a mean age of 53.7 ± 9.5 years, comprised of 19 females and 25 males, and a baseline weight and BMI of 102.1 ± 14.5 kg and 34.6 ± 4.9 kg/m^2^, respectively. Comprehensive demographic and clinical characteristics for all participants and the subgroup of high responders are provided in Table 1; Table S1.

**TABLE 1:** Anthropometric changes and cardiometabolic effects of 8‐week 40%–50% energy restriction in adults with prediabetes.

Variable mean (SD)	Pre‐intervention (*N* = 44)	Post‐intervention (*N* = 44)	*p*‐value Wilcoxon
Weight (kg)	102.1 (14.5)	88.1 (12.5)	< 0.001
BMI (kg/m^2^)	34.6 (4.9)	29.9 (4.3)	< 0.001
Waist circumference (cm)	117.0 (11.1)	104.4 (10.9)	< 0.001
Hip circumference (cm)	118.1 (11.7)	109.5 (11.0)	< 0.001
Fat mass (kg)	40.2 (9.7)	32.7 (9.6)	< 0.001
Fat‐free mass (kg)	60.3 (9.4)	54.2 (8.4)	< 0.001
Body fat (%)	39.7 (6.6)	37.2 (7.9)	< 0.001
Fasting plasma glucose (mmol/L)	6.4 (0.6)	5.6 (0.5)	< 0.001
Fasting insulin (mU/L)	13.9 (6.5)	8.1 (3.6)	< 0.001
C‐peptide (pmol/L)	1035.9 (329.2)	744.8 (286.5)	< 0.001
HOMA‐IR	3.9 (1.8)	2.1 (1.0)	< 0.001
HbA1c (%)	5.7 (0.4)	5.4 (0.3)	< 0.001
HbA1c (mmol/mol)	38.6 (4.0)	35.1 (3.5)	< 0.001
Total cholesterol (mmol/L)	5.0 (0.8)	3.8 (0.9)	< 0.001
HDL cholesterol (mmol/L)	1.2 (0.2)	1.0 (0.1)	< 0.001
LDL cholesterol (mmol/L)	3.1 (0.7)	2.2 (0.7)	< 0.001
Triglycerides (mmol/L)	1.7 (0.9)	1.1 (0.7)	< 0.001
Systolic blood pressure (mmHg)	130.2 (14.0)	114.3 (13.7)	< 0.001
Diastolic blood pressure (mmHg)	82.1 (10.4)	71.0 (7.4)	< 0.001
hs‐CRP (mg/L)	4.7 (5.2)	4.2 (5.6)	0.024

*Note:* Data expressed as mean ± SD. Statistical significance was calculated using the Wilcoxon matched‐pairs signed‐rank test. *N* = 44 participants. Significance levels are indicated as *p*‐values.

### 1.2 Energy‐Restriction‐Induced Weight Loss Reduces Circulating Extracellular Vesicle (cEV) Markers

To evaluate the effects of the energy‐restricted intervention on the proteome of both neat plasma and plasma enriched in circulating extracellular vesicles (cEV), we used untargeted liquid chromatography with tandem mass spectrometry (LC–MS/MS) (Figure 1A). While previous studies have used aptamer‐based SOMAscan technology to analyze proteome responses to energy restriction in neat plasma (Geyer et al. 2016), this study adds a new dimension by examining changes in the cEV proteome. EVs, lipid‐bound carriers essential for intercellular communication (Lark et al. 2024), are secreted by metabolically active tissues such as adipose, liver, pancreas, and muscle, making them highly diet‐responsive particles (Eitan et al. 2017; González‐Blanco et al. 2024) and linked to metabolic disorders such as insulin resistance and T2D (González‐Blanco et al. 2024; Akbar et al. 2019; Huang‐Doran et al. 2017). For example, liver‐derived EVs help regulate glycemic control and respond to hyperglycemia in mice (Miotto et al. 2024). In older adults with insulin resistance, intermittent fasting modulates neuron‐derived EVs to improve neuronal insulin signaling (Kapogiannis et al. 2024). To enrich cEVs, we used an immunoaffinity‐based enrichment protocol, yielding only a 24% overlap with proteins found in neat plasma (Figures S1 and S2). The Methods and Supporting Figures provide details on enrichment, quality control, and statistical analyses. Post‐filtration, we identified 1846 non‐redundant proteins in cEV‐enriched plasma and 570 in neat plasma, with 449 proteins overlapping.

To validate the enrichment of cEV‐associated proteins, we cross‐referenced our data with Vesiclepedia (www.microvesicles.org) (Chitti et al. 2024), a comprehensive EV protein database curated by the Mathivanan Lab (Melbourne, Australia). Of the proteins identified by LC–MS/MS, 96% (1779/1846) were recognized by Vesiclepedia, with 90% (1606/1779) annotated as EV‐associated (Figure 1B). Analysis using the FunRich (Functional Enrichment) tool (www.funrich.org) revealed that the top five locations of these EV‐associated proteins were the cytoplasm, exosomes, nucleus, lysosomes, and plasma membrane, confirming their expected absence in neat plasma (Figure S3). Furthermore, our dataset included 97 of the top 100 most frequently reported EV‐associated proteins, including key exosome markers CD63 and CD9 (Figure S4). Notably, 21 of the top 100 EV‐associated proteins (22%), including CD63, CD9, FLOT1, SDCBP, HSP90AA1, HSPA8, HSP90AB1, and FLOT2, showed significant decreases following the dietary intervention (Figure 1C; Figure S4). This reduction could be due to lower circulating EV numbers and/or smaller size, in alignment with previous studies showing that EV number correlates with body weight in adolescents with obesity (Kobayashi et al. 2024) and that EV size significantly decreases following weight loss (DiStefano et al. 2024). Even though our method does not allow for the identification of the tissue origin of cEVs, another study in obesity showed that cEVs are primarily derived from adipocytes, hepatocytes, B cells, and neurons (Kobayashi et al. 2024), suggesting that reduced cEV secretion and/or trafficking following weight loss may be linked to a decrease in fat mass. Additionally, the low‐energy diet may have influenced glucose uptake in muscle by downregulating FLOT1 (flotillin‐1), a membrane protein crucial for recruiting the glucose transporter GLUT4 to the sarcolemma in response to insulin (Fecchi et al. 2006). Overall, the findings suggest that a low‐energy diet significantly impacts circulating EV markers in individuals with prediabetes, which are key mediators of cell communication in metabolic disease (Huang‐Doran et al. 2017).

### 1.3 Energy‐Restriction‐Induced Weight Loss Reduces Markers of Protein Synthesis and Degradation

Following the intervention, we observed significant changes in cEV‐associated proteins: 185 proteins (10%) exhibited significant increases, while 280 proteins (15%) showed significant decreases (Figure 1D). Most of these proteins were annotated in Vesiclepedia, with 153 proteins increasing and 253 decreasing (Figure 1E). The heatmap in Figure 1F highlights the top 50 most significantly altered EV‐associated proteins based on *p*‐value.

To elucidate the functional implications of our findings, we performed gene set enrichment analysis (GSEA) on the differentially expressed (DE) proteins adjusted to a 5% false discovery rate (FDR) (adj *p*‐value < 0.05). GSEA identifies overrepresented protein groups across time points, yielding insights into the biological functions of the proteomics data. We observed a significant downregulation of key biological pathways related to protein synthesis and degradation, particularly mTORC1 signaling (adj *p* = 0.0072), the ubiquitin‐proteasome system, and ubiquitination (Figures 1G,H, 3A). The GSEA database for mTORC1 signaling included 200 genes compiled from five independent experimental datasets of rapamycin treatment and variable levels of mTOR expression (Jimenez et al., 2009; Düvel et al. 2010; Zeng et al. 2013; Rojo et al. 2014). mTORC1, a central regulator of cellular growth, is activated by growth factors and nutrients, with dysregulation linked with obesity and T2D, neurodegeneration, cancer, and accelerated aging (Panwar et al. 2023; Ma and Blenis 2009; Laplante and Sabatini 2012; Liu and Sabatini 2020). Upon activation, mTORC1 phosphorylates the translation initiation factor 4E‐binding protein 1 (4E‐BP1) along with S6 kinase 1 (S6K1), promoting protein synthesis while inhibiting pathways involved in endogenous antioxidant defense and DNA repair (Panwar et al. 2023; Ma and Blenis 2009; Laplante and Sabatini 2012; Liu and Sabatini 2020).

**FIGURE 2:**
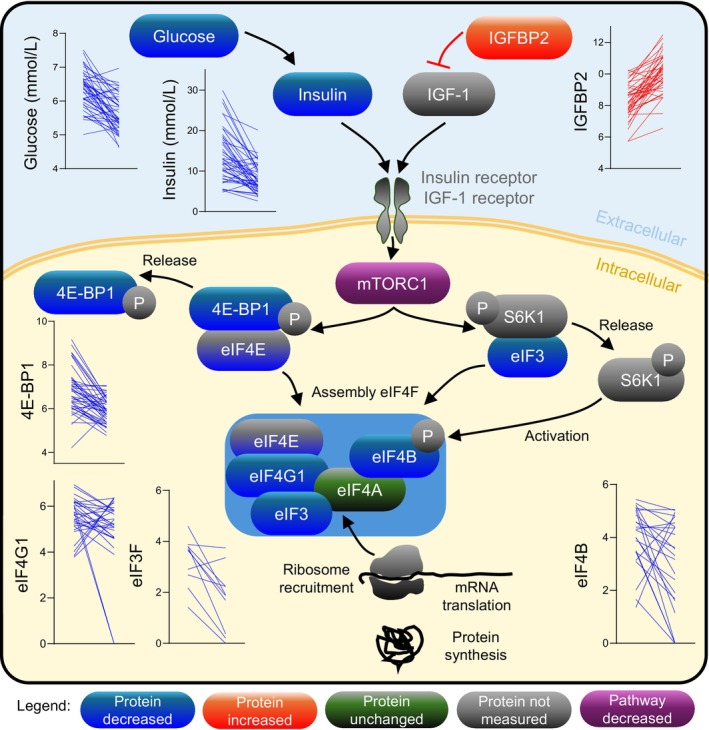
Energy‐restriction‐induced weight loss reduces markers of protein synthesis downstream of mTORC1. Diagram of the glucose/insulin/mTORC1/eIF4E/protein synthesis signaling pathway. For all graphs, the arrows represent protein levels for each study participant (*n* = 44) from baseline to post‐intervention. Increased levels are shown by increasing arrows in red, whereas decreased levels are shown by decreasing arrows in blue. All changes shown are significant (*p* and adj *p* < 0.05), except for eIF4E (*p* < 0.05 but adj *p* > 0.05). Glucose and insulin were measured and previously reported by the PREVIEW study. All other proteins were extracted from the LC–MS/MS proteomics dataset. Protein color represents decreased abundance (blue), increased abundance (red), not measured/not detected (gray), or statistically non‐significant (green). *P*, phosphorylation; eIF, eukaryotic translation initiation factor.

Following the energy‐restricted diet, levels of several proteins directly downstream of mTORC1 were reduced in cEVs, including eIF4G1 (adj *p* = 0.0234) and eIF4E (*p* = 0.0236, adj *p* = 0.0792), two essential components of the eIF4F complex; eIF4B (adj *p* = 0.0013), which is crucial for helicase activation; eIF3 subunits eIF3L, eIF3B, and eIF3F (adj *p* = 0.0026, 0.0003, and 3.24E‐06, respectively); and eIF2G (adj *p* = 0.0238), a member of the eIF2 complex (Figures 1D, 2). A visual representation of the mTORC1 pathway leading to protein synthesis is provided in Figure 2. These proteins are essential for (1) ribosome recruitment, (2) translation initiation, (3) elongation and translation itself, (4) mRNA recruitment, and (5) delivery of the initiator Met‐tRNAi, suggesting a broad and uniform reduction in the core translation machinery. Notably, 4E‐BP1 showed the greatest downregulation by fold change (FC = −0.87, adj *p* = 7.43E‐06) in the Olink dataset, which was independently obtained from the mass spectrometry data (Figure 4A,B). 4E‐BP1 is a translation inhibitor phosphorylated by mTORC1. Under nutrient‐rich conditions, mTORC1 phosphorylates 4E‐BP1 to release its inhibition on eukaryotic translation initiation factor 4E (eIF4E) and enable translation. Under nutrient scarcity, 4E‐BP1 remains unphosphorylated and actively inhibits eIF4E, and its expression decreases (Levy et al. 2024). This sequestration of eIF4E prevents the formation of the translation complex eIF4F. Other subunits essential for complex formation such as eIF4G, eIF4A, and eIFG1 (Böhm et al. 2021), together with eIF3, which recruits the 40S ribosomal subunit to initiate translation, were also significantly reduced. The coordinated assembly and activation of the eIF4F and eIF3 complexes are essential for ribosome recruitment and mRNA translation, leading to protein synthesis (Levy et al. 2024; Böhm et al. 2021; Majeed et al. 2021; Yi et al. 2017). In this study, the reduction in several subunits of translation initiation factors suggests decreased secretion and/or trafficking of proteins involved in protein synthesis. This effect might have been mediated by a diet‐induced reduction in insulin levels (13.9 ± 6.5 to 8.1 ± 3.6 mU/L, Table 1), as insulin directly interacts with eIF3 and regulates the association between eIF3 and eIF4G to promote protein synthesis (Levy et al. 2024).

**FIGURE 3:**
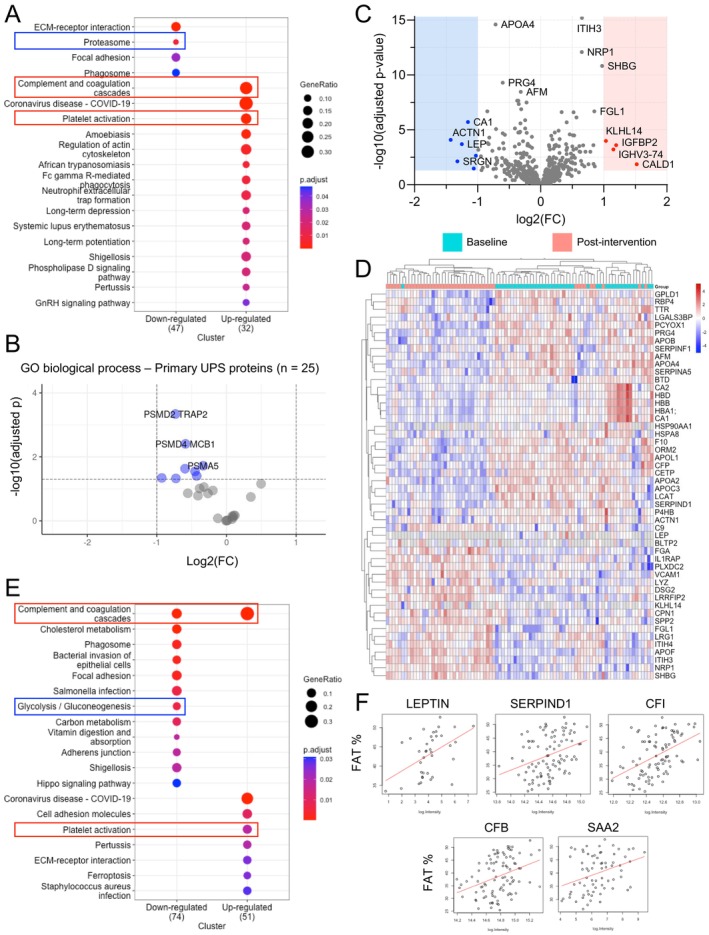
Energy‐restriction‐induced weight loss reduces markers of protein degradation via the ubiquitin‐proteasome system. (A) Top differentially expressed pathways from KEGG pathway enrichment analysis post‐CR. Input = cEV dataset. Adj *p*‐values are color‐coded. Pathways of interest are labeled (red rectangle = increased, blue rectangle = decreased). Larger circles represent higher protein ratios. (B) Volcano plot of proteins with the primary annotated “Gene Ontology (GO) Biological Process” as the ubiquitin‐proteasome system. *N* = 25 proteins. Input = cEV dataset. Significantly decreased proteins are labeled (blue, adj *p* < 0.05). No significantly increased proteins were detected. (C) Volcano plot of all proteins from the neat plasma dataset. *N* = 569 proteins (one protein, APOF, is out of the chart axis due to a large adj *p* = 6.49E‐21). Significantly decreased (blue, adj *p* < 0.05) and significantly increased (red, adj *p* < 0.05) with absolute FC > 2 are labeled. (D) Top 50 significantly differentially expressed neat plasma proteins by adj *p*‐value, paired. Each row represents a significantly decreased (blue) or increased (red) protein. Each column represents a study participant at baseline (teal) and at week 8 (pink). (E) The same KEGG analysis as per “A” with input = neat plasma dataset. Pathways of interest are labeled (red rectangle = increased, blue rectangle = decreased). (F) Scatter plots of selected proteins significantly correlated with both body fat % at baseline and with Δ fat % (fat % at post‐intervention minus fat % at baseline). Input = neat plasma dataset. Each dot represents a measurement of baseline fat % and respective protein level in a study participant. Red lines (best fit lines) with a positive slope represent a positive association between fat % and protein abundance. All reported significant changes are adj *p* < 0.05.

In addition, levels of ubiquitin‐proteasome system proteins responsible for degrading damaged or misfolded proteins (Li et al. 2022) were also reduced in cEVs, suggesting a potential decrease in secretion and/or trafficking of proteasomal proteins (Figures 1G,I, 3A). Previous studies, primarily in rodents, have shown that energy restriction can reduce proteasome activity, ubiquitination, and deubiquitination (Altun et al. 2010; Chen et al. 2019). In this study, following the intervention, pathway analyses using KEGGs, GSEA, and IPA revealed significant downregulation of the proteasome and all detected proteasomal proteins, together with inhibition of protein ubiquitination, deubiquitination, protein sorting, and protein folding (Figures 1G,I, 3A; Figure S5). To pinpoint the proteins behind this effect, we categorized the 1846 cEV proteins according to their Gene Ontology (GO) Biological Process annotations, identifying 25 involved in the ubiquitin‐proteasome system. Of these, 8 were reduced post‐intervention, including proteasome complex components with regulatory (PSMD2, PSMD4), catalytic (PSMA5, PSMA3, PSMB2), and ubiquitination‐facilitating (HECTD3) roles (Figure 3B). Taken together, the results indicate that energy restriction is linked to significantly reduced levels of proteins involved in both initiating protein synthesis and facilitating protein degradation in vesicle‐enriched plasma.

### 1.4 Energy‐Restriction‐Induced Weight Loss Lowers Inflammatory and Coagulation Markers

Beyond changes in pathways regulating protein synthesis and degradation, we observed a significant alteration in coagulation and platelet activation pathways following the intervention. Previous studies have shown that energy restriction improves coagulation profiles in obese mice (Lijnen et al. 2012) and hemostatic balance in humans (Morel et al. 2011), and it lowers platelet activation markers (Ezzaty Mirhashemi et al. 2021). Our study cohort comprised individuals with overweight/obesity who typically exhibit dysregulated thrombotic states due to excess adiposity, insulin resistance, and chronic inflammation (Van Gaal et al. 2006; Sebo et al. 2019; Samocha‐Bonet et al. 2008; Basili et al. 2006; Anfossi et al. 2009). Following the intervention, participants experienced a 13.7% average weight loss, a 7.5 kg reduction in fat mass (Table 1), and significant changes in proteins involved in the complement and coagulation cascade, both in cEV (Figures 1G, 3A; Figure S6) and neat plasma (Figure 3C–E). The neat plasma proteome revealed 113 increased proteins (20%) and 107 decreased proteins (19%) following the low‐energy diet; among these, multiple were complement and coagulation proteins (e.g., Complement Component C9, Complement Factor P, Factor 10, and SERPIND1) involved in inflammation, platelet activation, degranulation, aggregation, and fibrin clot formation (Figure 3E; Figure S7).

**FIGURE 4:**
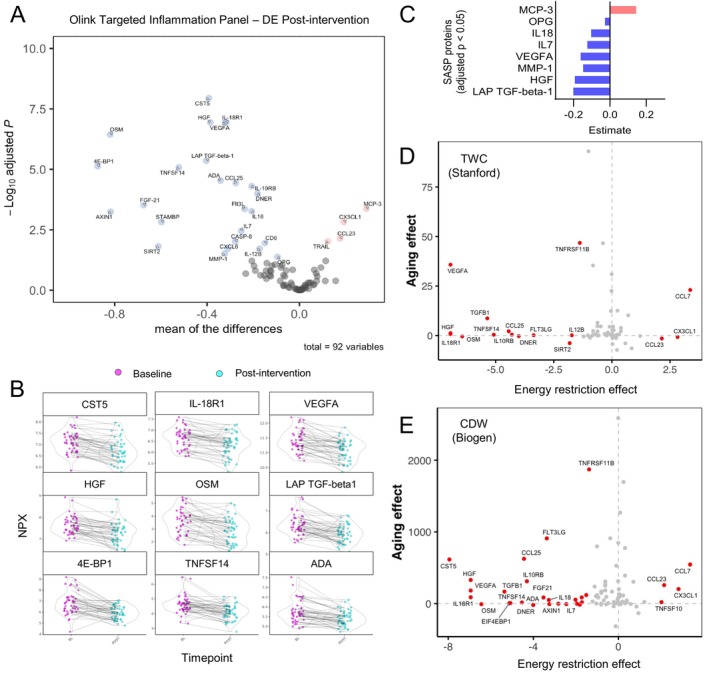
Energy‐restriction‐induced weight loss lowers senescence factors and reverses the aging effect of multiple inflammatory proteins. (A) Volcano plot of all proteins from the Olink Targeted Inflammation Panel. *N* = 92 proteins. Significantly decreased post‐intervention (blue, adj *p* < 0.05, *n* = 25 proteins) and significantly increased post‐intervention (red, adj *p* < 0.05, *n* = 4 proteins) are labeled. Top differentially expressed proteins by *p*‐value are named. (B) Individual abundances for the top 9 differentially expressed proteins by *p*‐value, paired. Each dot represents the protein abundance in each study participant at baseline and week 8. NPX, Normalized Protein Expression. (C) All significant SASP proteins (adj *p* < 0.05) from the Olink dataset that belong to the curated SASP list provided in Supporting Information. Significantly decreased (*n* = 7, blue) and significantly increased (*n* = 1, pink) SASP proteins. (D, E) Correlation of results from the Olink inflammation panel post‐intervention with the human plasma proteome across lifespan in two previously published datasets. Representation of the intervention effect (X axis, protein abundance changes post‐ER) and the calculated aging effect from TWC Stanford and CDW Biogen (Y axis, protein abundance changes with increasing age).

Given that reduced adiposity lowers inflammation, which is closely linked to thrombosis (Stark et al. 2021; Hildebrandt et al. 2022), it is plausible that the observed changes in coagulation and complement markers were associated with diet‐induced fat mass loss. Supporting this hypothesis, 24 of the 40 proteins significantly correlated with baseline fat percentage changed post‐intervention. These included leptin (a fat‐derived adipokine expected to decrease) along with multiple complement and coagulation proteins (e.g., SERPIND1, Complement Factor I, Complement Factor B) (Figure 3F; Figure S8). These findings establish a strong connection between diet‐induced fat loss and the reduction of complement and coagulation proteins associated with thrombosis and inflammation, underscoring the importance of reducing adiposity to lower inflammatory and coagulation markers. It is well known that long‐term energy restriction suppresses inflammation, lowering biomarkers such as CRP, TNF‐α, and IL‐6 (Geyer et al. 2016; Alfadda et al. 2014; Perry et al. 2023; Oller Moreno et al. 2018). To supplement the mass spectrometry data, we used a targeted cytokine assay (Olink Target Inflammation panel) covering 92 proteins involved in inflammation and senescence to investigate the impact of energy restriction on inflammatory proteins. Detailed methods and the list of proteins measured are provided in Methods, Figure S9, and Table S2. Out of 92 proteins analyzed, 75 were detected in over 75% of samples. Among these, 29 proteins (32%) exhibited significant changes following the intervention (adj *p* < 0.05), with 25 downregulated and 4 upregulated proteins (Figure 4A), indicating an overall reduction in inflammatory markers. The most significant downregulated proteins based on *p*‐value are shown in Figure 4B. Significant reductions in immune‐related cytokines, such as IL‐12B, IL‐18, IL‐10RB, and IL‐7, suggest a shift toward reduced inflammation and potentially lower risk of autoimmune conditions. These findings align with extensive research in animal models, which demonstrates the broad protective effects of energy restriction against autoimmune diseases (Fernandes 2008). In addition, circulating levels of chemokines CCL25 and CXCL6 were significantly reduced, both of which are involved in immune cell trafficking and inflammatory regulation. Several growth factors related to inflammation and tissue remodeling were also affected, including Flt3L (hematopoiesis), HGF (hepatocyte regeneration), VEGFA (angiogenesis), and FGF‐21 (metabolic regulation and tissue repair). These factors are essential to immune function, tissue regeneration, and metabolic processes, highlighting how energy restriction orchestrates the intricate crosstalk between metabolic and immune‐inflammatory pathways. Other inflammation‐related proteins also showed marked reductions, including metalloproteinases (e.g., MMP‐1), protease inhibitors (e.g., CST5), pro‐apoptotic proteins (e.g., CASP‐8, LAP TGF‐β‐1), and immune regulators (e.g., OPG, CD6, TNFSF14). These findings emphasize the extensive anti‐inflammatory effects of energy restriction, evidenced by the downregulation of a broad spectrum of inflammatory‐related cytokines and proteins, going beyond the typical reduction in high‐sensitivity C‐reactive protein, which decreased from 4.7 to 4.2 mg/L over the 8‐week study period (*p* = 0.024) (Table 1).

### 1.5 Energy‐Restriction‐Induced Weight Loss Lowers Senescence Factors and Reverses the Aging Effect of Multiple Proteins

Building on the observed reduction in multiple cytokines and chemokines (Figure 1A) and informed by our previous findings showing reductions in p16, IL6, IL1a, and MMP9 transcripts in colon mucosa samples from individuals practicing chronic energy restriction (Fontana et al. 2018), we hypothesized that the intervention may also reduce circulating components of the SASP. A previous study on moderate energy restriction in healthy young‐to‐middle‐aged individuals enrolled in CALERIE found reduced concentrations of various senescence biomarkers in both plasma and adipose tissue (Aversa et al., 2024). SASP factors are secreted by senescent cells, which are characterized by a stable, typically irreversible cell cycle arrest driven mainly by the cyclin‐dependent kinase (CDK) inhibitors p16 and p21 (Hernandez‐Segura et al. 2018; Basisty, Kale, Jeon, et al. 2020; Basisty, Kale, Patel, et al. 2020). Extensive research has shown that senescent cells and the SASP contribute to aging and numerous age‐associated pathologies (Chaib et al. 2021, 2022; Martínez‐Zamudio et al. 2021; Yousefzadeh et al. 2021; Khosla et al. 2020), establishing cellular senescence and its secretory profile as a core aging mechanism (López‐Otín et al. 2023). To investigate this, we curated a comprehensive list of 163 SASP factors from three key sources: the Senescence‐Associated Secretory Phenotype (Campisi lab, 2010), the SenMayo list of senescence‐associated genes (2022), and the SenNet guidelines for detecting senescence (2024). Of these 163 SASP targets, 30 were in our Olink inflammation panel (Figure S10; full list in Table S3). Seven of these plasma proteins (VEGFA, HGF, LAP, TGF‐beta‐1, IL18, IL7, MMP‐1, and OPG) were significantly reduced following the intervention, while one protein (MCP‐3) was upregulated (Figure 4C). Upon age stratification, the decreasing trend in SASP factors was also observed in older participants (> 55.5 years, median age) (Figure S10), suggesting that older populations can also benefit from systemic reduction in several SASP factors.

Next, we examined whether energy restriction could reverse age‐related changes in protein levels within the Olink inflammation panel. Using two publicly available datasets (TWC Stanford and CDW Biogen, Methods), which included plasma protein levels from approximately 54,000 individuals spanning the human lifespan, we quantified the impact of aging on these circulating proteins. We then compared these age‐related changes to the effects of the intervention in our cohort by plotting the impact of aging on the Y axis (increasing Y represents higher protein levels with age) and the dietary effect on the X axis (increasing X represents higher protein levels post‐intervention) in a scatter plot. Several proteins that increase with age (those with Y > 0), including TNFRSF11B, FLT3LG, CCL25, CST5, VEGFA, TGFB1, HGF, IL10RB, and IL18, were significantly reduced after energy restriction (X < 0), suggesting a reversal of age‐associated changes. Notably, TNFRSF11B (also known as osteoprotegerin, a regulator of bone metabolism) and VEGFA (Vascular Endothelial Growth Factor A, a regulator of angiogenesis) showed the most pronounced improvements (Figure 4D,E). The age‐reversal effect was also observed in individuals over 55.5 years of age (the median age in this cohort) (Figure S10), with only one protein, CCL23, exhibiting a pro‐aging effect. This suggests that improvements in protein levels can occur even in older individuals, highlighting the potential for age‐related changes to be mitigated through energy restriction. Collectively, these findings suggest that the energy‐restricted intervention not only decreased inflammatory and SASP factors but also reversed age‐related changes in multiple proteins, akin to the improvement observed in the age‐associated inflammatory marker sICAM‐1 with alternate‐day fasting (Stekovic et al. 2019).

### 1.6 A Universal Signature of Energy Restriction in Humans

This and other studies (Geyer et al. 2016; Kapogiannis et al., 2024; Alfadda et al. 2014; Perry et al. 2023; Oller Moreno et al. 2018; Stekovic et al. 2019; Beals et al. 2023) contribute to establishing a consistent, reproducible, and universal proteomics signature of energy restriction in humans. This is crucial for comparing intervention effects across studies and examining the similarities and differences between human and animal models. Key features of energy restriction include elevated levels of liver‐produced Sex Hormone‐Binding Globulin (SHBG, log(FC) = 1.70, adj *p* = 4.63E‐11), which regulates the availability of estrogen and testosterone; Insulin‐Like Growth Factor‐Binding Protein 2 (IGFBP‐2, log(FC) = 1.53, adj *p* = 4.90E‐11), which modulates IGF‐1 availability; and apolipoprotein F (APOF, log(FC) = 0.61, adj *p* = 6.49E‐21), an inhibitor of cholesteryl ester transfer protein activity on LDL. These changes are commonly accompanied by reductions in weight and fat mass (Table 1), decreased leptin levels (log(FC) = −1.25, adj *p* = 0.0002), increased adiponectin (log(FC) = 0.1914, adj *p* = 0.0389), and reductions in cholesterol, apolipoprotein A4 (APOA4, log(FC) = −0.7155, adj *p* = 2.55E‐15), and apolipoprotein B (APOB, log(FC) = −0.3474, adj *p* = 4.45E‐08), biomarkers commonly associated with metabolic syndrome (Figures 1D–F, 3C,D; Figure S11). At the pathway level, energy restriction alters cholesterol metabolism, glycolysis/gluconeogenesis, and phospholipase signaling and activity, aligning with the increased reliance on lipid utilization as a primary energy source (Figure 3A,E; Figures S5–S7). These changes are frequently associated with significant reductions in fasting blood glucose levels, as reported in the PREVIEW and other energy restriction studies (Lean et al. 2018; Raben et al. 2021).

However, not all individuals showed improved glucose responses to the intervention, even with strict adherence, as evidenced by significant weight loss (Figure S11). To better understand this variability and identify individuals likely to experience glycemic improvement, we developed a predictive model to forecast the fasting blood glucose response to a low‐energy diet. While previous PREVIEW substudies have utilized metabolomics (Relva et al. 2024) and lipidomics (Jiang et al. 2024) to develop predictive biomarker panels, this is the first substudy to employ a proteomics‐based approach. Thirty‐one (*n* = 31) proteins from neat plasma collected at baseline were significantly correlated (*p* < 0.05) with ∆ glucose (defined as fasting glucose post‐intervention minus fasting glucose at baseline, in mmol/L). Proteins derived from cEVs were not included due to their limited feasibility in clinical practice, and proteins with missing values (*n* = 14) were removed. The remaining 17 proteins were included in a multivariate linear regression analysis. Stepwise reduction regression yielded a significant predictive panel for ∆ glucose consisting of six proteins (*F*_(6,37)_ = 5.927, *p* < 0.00021), explaining 49.01% of the variance (*R* ^2^ = 49.01%; adjusted *R* ^2^ = 40.74%), with a residual standard error of 0.50 mmol/L. To enhance the model, 14 readily available clinical variables were added to a second multivariate linear regression analysis, in addition to the 17 proteins. The new model yielded a significant predictive panel for ∆ glucose consisting of 7 proteins and 8 clinical variables (*F*_(15,28)_ = 6.39, *p* < 0.0000032), explaining 79.83% of the variance (*R* ^2^ = 79.83%, adj *R* ^2^ = 69.02%), with a residual standard error of 0.36 mmol/L. The 15‐marker integrated panel included OGTT blood glucose at 30 min, total cholesterol, CRP, ALT, AST, fasting blood glucose, insulin, C‐peptide, platelet glycoprotein Ib alpha chain (GP1BA), retinoic acid receptor responder protein 2 (RARRES2/Chemerin), inter‐alpha‐trypsin inhibitor heavy chain H1 (ITIH1), immunoglobulin heavy constant gamma 1 (IGHG1), properdin (CFP), phosphatidylcholine‐sterol acyltransferase (LCAT), and immunoglobulin kappa joining 1 (IGKJ1) (Table S4). Among these, insulin, chemerin, and LCAT are recognized as key regulators of glucose metabolism and cholesterol homeostasis, respectively, and are classified as proteins of interest in the Human Diabetes Proteome Project (https://hupo.org/Human‐Diabetes‐Proteome‐Project) together with GP1BA. Therefore, the results support the use of predictive models to enable personalized, patient‐centered care, achieving 80% accuracy in predicting glucose response.

## 2 Discussion

The metabolic state of prediabetes is linked to accelerated organ aging in humans, and it can be reversed with an energy‐restricted diet (Dove et al. 2024; Tabák et al. 2012; Schlesinger et al. 2022; Cai et al. 2020; Galaviz et al. 2022; Beals et al. 2023; Nikparast et al. 2024). In this study, we assess the impact of a low‐energy diet on both the neat plasma and vesicle‐enriched plasma in adults with prediabetes, using both hypothesis‐driven (targeted) and hypothesis‐free (untargeted) proteomics. We demonstrate that a 12% weight loss resulting from 8 weeks of severe energy restriction is associated with significant changes in markers of inflammation, coagulation, senescence, protein synthesis, and protein degradation, suggesting improved health at the molecular level. The reductions in extracellular vesicle markers may stem from decreased secretion and/or trafficking, potentially linked to the shrinkage of adipose tissue and rewiring of liver metabolism. The findings are consistent with previous studies on extracellular vesicles during weight loss, which have been linked to the pathophysiology of metabolic diseases (González‐Blanco et al. 2024; Akbar et al. 2019; Huang‐Doran et al. 2017; Kobayashi et al. 2024; DiStefano et al. 2024). Of particular interest is the downregulation of mTORC1 protein targets involved in protein synthesis, a crucial aging pathway that integrates nutrient signaling, disease, and lifespan and is often dysregulated in metabolic diseases (Cochemé and Gil 2024; Panwar et al. 2023; Laplante and Sabatini 2012; Liu and Sabatini 2020; Koundouros and Blenis 2022). Furthermore, levels of FGF21, a liver‐derived hormone often elevated in metabolic disorders such as obesity and insulin resistance (Qian et al. 2022; Lewis et al. 2019; Dostálová et al. 2009), were also reduced post‐intervention. This reduction suggests that energy‐restriction‐induced weight loss, particularly through reductions in liver fat, likely improved FGF21 sensitivity and resulted in improved metabolic health. Cardiometabolic improvements have been previously reported in individuals with metabolic syndrome following 12 weeks of time‐restricted eating (Wilkinson et al. 2020), suggesting a potential basis for combining temporal and caloric restriction strategies to optimize metabolic health outcomes. In addition, lower inflammatory and senescence factors following the dietary intervention likely contribute to reductions in chronic inflammation (Cochemé and Gil 2024; Tizazu 2024), further improving overall health, as cellular senescence contributes to aging and various pathologies via secretion of pro‐inflammatory factors as part of its hypersecretory phenotype (Chaib et al. 2021, 2022; Martínez‐Zamudio et al. 2021; Yousefzadeh et al. 2021; Khosla et al. 2020).

Our findings build upon and expand upon numerous studies on energy restriction. Previous studies of adipose tissue in overweight/obesity were limited by proteomic coverage (≤ 40 proteins), localized expression (adipocytes only), and small sample sizes (*n* = 8 participants) (Alfadda et al. 2014; Bouwman et al. 2009, 2014). Nevertheless, they reported energy‐restriction‐induced changes in glucose metabolism and β‐oxidation that align with our findings, including the downregulation of glycolysis/gluconeogenesis, carbon metabolism, and cholesterol metabolism, suggesting that adipose tissue and plasma reflect consistent metabolic shifts.

Our results also add weight to the well‐known effect of energy restriction on inflammation and inflammaging (Tizazu 2024; Franceschi and Campisi 2014). A previous study demonstrated that 3–6 months of energy restriction reduced CRP levels (Alfadda et al. 2014), a clinically validated marker of systemic inflammation. This result is noteworthy because chronic inflammation, known as “inflammaging,” is associated with aging and age‐related diseases (Cochemé and Gil 2024; Franceschi and Campisi 2014).

Recent advancements in protein detection, including Olink assays, SOMAscan, and mass spectrometry, now enable a deep and comprehensive understanding of the molecular changes induced by dietary interventions. For instance, the Weight Loss Maintenance study in the United States (2023) used the Olink Immune‐Oncology panel to uncover inflammatory proteins that significantly changed with weight loss (Perry et al. 2023), revealing considerable overlap with our findings, including changes in IL‐18, VEGFA, CASP‐8, TNFSF14, and HGF. Similarly, a 2016 Danish weight loss study identified changes in adipocyte‐secreted SERPINF1 and apolipoproteins, along with reductions in inflammatory markers such as CRP (Geyer et al. 2016), as also seen herein. The European DiOGenes study (2018) of non‐diabetic individuals with overweight/obesity on a low‐energy diet revealed significant changes in key proteins previously associated with weight loss, including SHBG, adiponectin, CRP, and IGFBP‐2 (Oller Moreno et al. 2018). This unique energy‐restriction‐specific proteomic signature was also observed in our study, emphasizing a consistent and universal response to energy restriction. Lastly, another energy‐restriction study in individuals with prediabetes and obesity revealed significant changes in autophagy pathways and mitochondrial proteins (Beals et al. 2023). Autophagy and the ubiquitin‐proteasome system (UPS) are the two pathways responsible for protein degradation in eukaryotic cells (Li et al. 2022; Lilienbaum 2013). In this study, we observed a downregulation of secreted and/or trafficked UPS proteins in response to energy restriction, potentially serving as a mechanism to preserve proteins in the context of reduced protein synthesis. This premise is consistent with the observed downregulation of secreted and/or trafficked mTORC1 targets following the intervention, which are the main promoters of protein synthesis in response to nutrient sensing (Panwar et al. 2023). Importantly, mTORC1 inhibition has been associated with extended lifespan in multiple model organisms (Robida‐Stubbs et al. 2012; Zhang et al. 2014; Harrison et al. 2009) and the mitigation of premature aging in hematopoietic stem cells (Chen et al. 2009), suggesting that lower intercellular transfer of mTORC1 targets during energy restriction may confer healthspan benefits.

### 2.1 Strengths and Limitations

Strengths of this study include a well‐defined and thoroughly characterized cohort (PREVIEW) with closely monitored dietary adherence, along with clinical data and longitudinally paired samples that enable robust intra‐individual comparisons. Experimentally, key strengths include the enrichment of extracellular vesicles, high‐dimensional proteomics via mass spectrometry, and targeted assays to detect inflammatory proteins often missed by mass spectrometry. Limitations include the absence of a control group, such as participants who underwent the intervention without significant weight loss or participants who did not undergo an intervention, restricting the findings to a pre‐post comparison. The study also cannot separate the effects of energy restriction from weight loss, as the intervention was designed for rapid weight reduction. The ≥ 12% weight loss threshold, chosen to maximize statistical differences, limits generalizability to individuals with lesser weight loss. Additionally, the 8‐week duration may be too short to capture long‐term effects on aging and chronic disease prevention. The study also does not compare continuous energy restriction to alternative approaches like intermittent fasting. Lastly, while the proteomic analysis is extensive, samples from other tissues (e.g., liver, muscle, adipose) would strengthen the findings.

## 3 Conclusion

Energy‐restriction‐induced weight loss in adults with overweight/obesity and prediabetes significantly reduced plasma biomarkers of inflammation, senescence, and metabolic dysfunction within 8 weeks, reversing several age‐related changes. The intervention was also linked to reduced intercellular transfer of mTORC1 targets, suggesting decreased protein synthesis. These molecular changes coincided with significant improvements in cardiometabolic health markers, underscoring the potential of energy restriction to enhance both metabolic and molecular health in prediabetes.

## 4 Materials and Methods

### 4.1 Participants

Adults with overweight/obesity (BMI ≥ 25 kg/m^2^; age 25–70 years) and prediabetes, defined by American Diabetes Association criteria (Committee et al. 2024) (fasting plasma glucose levels of 5.6–6.9 mmol/L and/or 2‐h glucose levels of 7.8–11.0 mmol/L after a 75‐g glucose challenge), were recruited for the PREVIEW study (clinicaltrials.gov NCT01777893) (Kahlert et al. 2016; Fogelholm et al. 2017). This 3‐year, randomized, multinational trial aimed to compare four dietary and exercise regimens for preventing diabetes in adults with prediabetes, following substantial weight loss through a very low‐energy diet (810 kcal or 3400 kJ per day). Between 2013 and 2015, 151 eligible participants were enrolled at the University of Sydney. The trial began with a standardized 8‐week weight reduction phase, during which participants were encouraged to achieve at least an 8% reduction in body weight before being randomized into the four investigational regimens. Upon completing this weight reduction phase, participants had lost between 6.2% and 20% of their body weight. Comprehensive characterization of the participants has been previously reported (Della Corte et al. 2023; Buso et al. 2021; Meroni et al. 2020) and is summarized in Table S1. The main findings of the PREVIEW study have also been published (Raben et al. 2021). For the current analysis, which focused on detecting proteomic and inflammatory changes linked to marked energy‐restriction‐induced weight loss, we selected participants who lost more than 12% of their initial body weight (*n* = 48). This threshold was chosen because previous research suggests that a weight loss of at least 10%–12% is associated with substantial improvements in cardiometabolic health and remission of T2D (Lean et al. 2018). By focusing on this group, we aimed to capture the most pronounced biological adaptations related to energy restriction and their implications for prediabetes biology and management. Moreover, by limiting the analysis to individuals who achieved greater than 12% weight loss, we have reduced variability in the data, allowing for clearer detection of the effects of energy restriction on proteomic and inflammatory markers. Outliers based on age (*n* = 1) and participants with unavailable samples (*n* = 3) were excluded, resulting in a final analysis cohort of 44 participants, whose details are provided in Table 1. All participants provided written informed consent to join the PREVIEW trial and the subsequent IRB‐approved substudies.

### 4.2 Intervention

The weight reduction protocol consisted of a very low‐energy diet (810 kcal or 3400 kJ per day) using nutritionally complete meal replacements from the Cambridge Weight Plan (Liverpool, UK) provided to the participants free of charge. Meals were sachets of soup, shakes, and porridge (4 sachets daily) to be dissolved in low‐fat milk (≤ 300 kcal/day) and water, fulfilling the daily requirements for vitamins and minerals. Meals provided 15.1% of energy from fat, 43.7% from protein (~85 g daily), and 41.2% from carbohydrates. Fiber content was 13.3 g/day, and additional consumption of *psyllium* fiber was encouraged. In addition to the sachets, up to 375 g of low‐starch vegetables such as tomatoes, cucumber, and lettuce were permitted daily (approximately ≤ 100 kcal). Other foods were not allowed. The diet, consumed for 8 weeks, provided up to 1210 kcal/day (including milk and optional vegetables), resulting in approximately 40%–50% energy restriction compared to the average Recommended Dietary Intake for males over 50 years (2300 kcal) and females over 50 years (1900 kcal) (National Research Council, Commission on Life Sciences, and Subcommittee on the Tenth Edition of the Recommended Dietary Allowances. “Recommended dietary allowances.” (1989)).

### 4.3 Blood Collection

For this study, blood samples were collected at two time points: baseline (pre‐intervention) and after 8 weeks of intervention (post‐intervention) via cannulation and venipuncture, respectively, with participants in a seated position, following a 10–12 h overnight fast. Blood was drawn into VenoSafe 10/9 mL EDTA tubes (VF‐109SDK) and centrifuged at 1500 G for 10 min. Plasma was carefully pipetted above the buffy coat, aliquoted into 1 mL vials, and immediately frozen at −80°C. The samples were maintained in a frozen state and thawed prior to analysis.

### 4.4 Olink Target Inflammation Panel

Protein biomarkers were quantified in multiplex using the Proximity Extension Assay (PEA) at the WEHI Genomics facility, using the 96 Olink Target Inflammation panel (Olink Proteomics, Sweden). The panel includes 92 protein assays and 4 internal controls. For this assay, plasma was taken from the same vials used in mass spectrometry, with all samples thawed simultaneously. Briefly, 1 μL of each plasma sample per panel was incubated with a cocktail of paired oligo‐labeled detection antibodies, and DNA extension was performed to create a specific barcode per interaction (Goldman et al. 2024). These barcodes were detected on a 96.96 Protein Expression IFC using a Biomark HD with HX controller (Fluidigm/Standard BioTools, USA). Detection cycle threshold [C(t)] values were then processed to NPX (Normalized Protein eXpression) values by normalizing to the internal Extension Control and the external Inter‐plate Control using Olink Signature software and the OlinkAnalyze R package (Lean et al. 2018). The values are expressed in NPX units on a log_2_ scale, while delta‐NPX indicates the difference between paired longitudinal samples. For the Olink inflammation panel, differential analysis of protein levels between pre‐ and post‐ER time points was conducted using paired t‐tests. To control for the risk of false positives due to multiple comparisons, the Benjamini‐Hochberg procedure was applied to adjust the *p*‐values.

### 4.5 Proteomics Sample Preparation

For the circulating extracellular vesicle (cEV) fraction, 200 μL of plasma was incubated with 15 μL ExoNET magnetic beads (INOVIQ, Australia) in a protein Lo‐Bind deep‐well plate (Eppendorf). After 20 min of incubation, plasma was removed, and beads were washed 3 times with 500 μL PBS. cEVs were eluted from the beads in two 15‐min elutions, where 50 μL of 1% SDC, 0.5% IGEPAL were added to the beads. The eluates were pooled in a fresh protein Lo‐Bind deep‐well plate, and lysis was performed in a final buffer composition of 1% SDS lysis buffer with 10 mM Tris (2‐Carboxyethyl) phosphine (TCEP) and 40 mM 2‐chloroacetamide (2‐CAA) and reduced/alkylated for 10 min at 95^0^. For plasma, 2 μL of untreated neat plasma was lysed, reduced/alkylated in 98 μL of the same buffer, from which an aliquot equivalent to 20 μg of protein was transferred to a protein Lo‐Bind deep‐well plate. Both sample preparations were then processed using the USP3 protocol (Dagley et al. 2019), with 20 μL of Magnetic PureCube Carboxy agarose beads (CubeBiotech) added to each sample, followed by on‐bead precipitation in acetonitrile (ACN, 70% v/v) for 20 min at RT using the ThermoMixer C (Eppendorf) at a mixing frequency of 400 rpm. Samples were placed on a magnetic rack, and three 200 μL washes were performed: twice with 70% ethanol and one 100% ACN. After washing, all traces of solvent were evaporated using a CentriVap (Labconco) before the addition of digestion buffer (100 mM Tris–HCl, pH 8) containing Lys‐C (Wako, 129–02541) and SOLu‐Trypsin (Sigma Aldrich, EMS0004) both at a 1:50 enzyme‐to‐protein ratio, and digestion was performed for 1 h at 37°C with a mixing frequency of 400 rpm. Digests were desalted using C18 stage tips (AffiniSep) as previously described (Rappsilber et al. 2007), vacuum centrifuged to dryness, and peptides were reconstituted in 0.1% formic acid, 2% ACN in preparation for liquid chromatography with tandem mass spectrometry (LC–MS/MS) analysis. The total plasma volume used in the analysis was normalized, allowing for accurate measurements of EVs per microliter.

### 4.6 Mass Spectrometry Analysis

Peptides (200 ng) were separated on a C_18_ fused silica column (inner diameter 75 μm, OD 360 μm × 15 cm length, 1.6 μm C18 beads) packed into an emitter tip (Aurora Elite, IonOpticks) using a custom nano‐flow high‐performance liquid chromatography (HPLC) system (Thermo Ultimate 300 RSLC Nano‐LC, PAL systems CTC autosampler). The HPLC was coupled to a timsTOF Pro (Bruker) equipped with a CaptiveSpray source, where peptides were loaded directly onto the column at a constant flow rate of 600 nL/min with buffer A (99.9% Milli‐Q water, 0.1% FA) and eluted with a 30‐min linear gradient from 2% to 34% buffer B (90% ACN, 0.1% FA). The timsTOF Pro (Bruker) was operated in diaPASEF mode using Compass Hystar 5.1. The settings on the TIMS analyzer were as follows: Lock duty cycle to 100% with equal accumulation and ramp times of 100 ms and 1/K0 start 0.6 V·s/cm^2^ end 1.6 V·s/cm^2^, capillary voltage 1400 V, dry gas 3 L/min, dry temp 180°C. The DIA methods were set up using the instrument firmware (timsTOF control 2.0.18.0) and included two windows in each diaPASEF scan across 16 × 25 m/z precursor isolation windows (32 windows) defined from *m/z* 400 to 1200, with 1 Da overlap, and CID collision energy ramped stepwise from 20 eV at 0.8 V·s/cm^2^ to 59 eV at 1.3 V·s/cm^2^.

### 4.7 Proteomic Database Searching, Pre‐Processing, and Normalization

Database searching of proteomic data files was performed using Spectronaut software (v.18) against the human‐reviewed databases (UniProt downloaded July 2022) using the BGS standard settings. Cysteine carbamidomethylation was included as a fixed modification, and N‐terminal acetylation and methionine oxidations were included as variable modifications. The FDR and PEP cutoff were set to less than 1% at the peptide and protein levels, and a minimum length of seven amino acids for peptides was specified. Enzyme specificity was set as C‐terminal to arginine and lysine for trypsin proteases with a maximum of two missed cleavages. Peptides were filtered based on the following criteria: only peptides with a Run‐Wise Posterior Error Probability (PG.PEP) of ≤ 0.05 that were also designated as proteotypic were retained. Protein intensities were estimated using the sum of the top three most abundant peptides. To address potential skewness in intensity data, log transformation (base 2) was applied to all protein intensity measurements. Subsequently, data normalization was performed using RUVIIIC methods to correct for systematic technical variations across the experimental runs. The mass spectrometry proteomics data have been deposited to the ProteomeXchange Consortium via the PRIDE (Goldman et al. 2024) partner repository with the dataset identifier PXD056694.

### 4.8 Statistical Analysis of Proteomics Data

Multivariate analysis, including principal component analysis (PCA), was employed to identify any potential outliers. Pairwise comparisons and ANOVA were conducted using the limma package (v. 3.60.3) in R. Adjustments for multiple comparisons were applied using the Benjamini‐Hochberg procedure to control the FDR. A protein was deemed significantly differentially expressed if its FDR was 5% or lower. A correlation test was performed to identify the strength and direction of the association of proteins with differences in various variables, such as weight and BMI, using the Pearson method. Functional enrichment analysis was performed on the sets of differentially expressed proteins to identify overrepresented biological pathways and functions. This analysis utilized databases such as Kyoto Encyclopedia of Genes and Genomes (KEGG), Reactome, and Gene Ontology (GO), employing the clusterProfiler (v. 4.12.0) R package. Visualization of the data and the results of the analyses was performed using ggplot2 (v. 3.5.1) and Spotfire (Tibco).

### 4.9 SASP Analysis of Olink Target Inflammation Panel

To evaluate the effect of ER on cellular senescence, we focused on SASP proteins. These proteins were identified from the inflammation panel based on previously established markers.^[1–3]^ Their changes in response to ER were visualized and labeled using a volcano plot. Given the accumulation of senescence with advancing age, an age‐stratified analysis by restricting the dataset to participants older than the median age of 55.5 years was also performed.

### 4.10 GSEA of Untargeted Proteomics

For the untargeted proteomics data, GSEA was performed using the clusterProfiler v4.4.4.^[4]^ package in R. Proteins were ranked by a combined metric, calculated as the product of ‐SignedLog2 fold change (FC) and the Log_10_ of the adj *p*‐value. GSEA was employed to identify biological pathways and processes significantly enriched in response to CR. Pathways were sourced from the KEGG, Hallmark gene sets, and Reactome databases.^[5]^ A significance threshold of a Benjamini‐Hochberg‐adj *p*‐value < 0.05 was applied.

### 4.11 Comparison of ER and Aging Effects on Plasma Protein Levels

We explored whether ER reverses the aging‐related changes in plasma protein levels. For this analysis, two publicly available datasets (CDW and Stanford) were used, each of which contained approximately 3000 plasma proteins.^[6,7]^ These datasets included samples from approximately 4000 and 50,000 participants across the lifespan, respectively. Protein abundances from both datasets were log‐transformed, and multivariable linear regression was used to calculate the aging effect based on the provided coefficients (fold changes) and *p*‐values [aging effect = significant (coefficient of aging) * log_10_(*p*‐value)]. Then, the aging effect was compared with the corresponding ER effect. A scatter plot was used to visualize the relationship between ER and aging effects on the same proteins. On the Y axis, a positive aging effect indicates that the protein levels increased with aging. On the X axis, a positive value signifies that the protein levels increased following ER. Therefore, proteins located in the upper‐left quadrant are those that increased with aging (based on the CDW/Stanford dataset) but decreased following ER (according to our dataset).

### 4.12 FunRich Analysis of Cellular Component GO Database

The annotated cellular components of cEV proteins and their overlap with the Vesiclepedia database were analyzed using FunRich software (FunRich:: Functional Enrichment Analysis Tool:: Home, accessed July 2024) (Pathan et al. 2017). The cEV proteomics dataset, consisting of 1846 proteins, was used as input. Proteins were identified using UniProt Protein Accession identifiers, resulting in the recognition of 1843 proteins, which were subsequently mapped to 1779 unique genes. Among these, 1697 genes were present in the Gene Ontology (GO) Cellular Component database. The predominant cellular locations of cEV proteins are provided in the Supporting Information, with some proteins annotated in 2 or more locations.

### 4.13 Ingenuity Pathway Analysis

Alterations in canonical pathways were generated with IPA software (QIAGEN Inc., https://www.qiagenbioinformatics.com/products/ingenuity‐pathway‐analysis, accessed July 2024) using the cEV proteomics dataset as an input (*n* = 1846 proteins), including protein identifiers (Uniprot), fold changes (post‐CR/baseline), and adjusted *p*‐values. IPA identified 464 analysis‐ready molecules based on adj *p* < 0.05 and performed Core Analysis Expression Analysis based on log2(FC) values to identify significantly upregulated and downregulated canonical pathways based on Z score. An extensive list of significantly enriched pathways is presented in the Supporting Information.

### 4.14 Statistical Analysis

All statistical analyses were conducted using R 4.2.1 and 4.4.1. The ggpubr 0.6.0 package was used for the Olink visualizations.^[9]^ Unless otherwise specified, a significance level of adj *p*‐value < 0.05 was used for all analyses. To develop the predictive model, data normality of log‐transformed neat plasma proteins was tested with KS using the nortest package (v 1.0–4), and univariable analyses were performed using Pearson's (*n* = 384 normally distributed proteins) and Spearman's (*n* = 186 non‐normally distributed proteins) correlations with ∆ glucose (∆ glucose = post minus pre, in mmol/L). ∆ glucose was the response variable, and baseline protein levels were the predictor variable. Estimates (coefficients), Spearman rho, standard error, t‐value, and *p*‐value were extracted for each protein analyzed. *p*‐value adjustments for multiple comparisons were applied using the Benjamini‐Hochberg method, with significant differential expression considered for FDR < 5%. Significant proteins (*n* = 31, non‐adjusted *p* < 0.05) were selected for multivariable linear regression with stepwise reduction to generate a predictor panel for change in fasting blood glucose following CR. After removing proteins with missing values in one or more participant samples (*n* = 14), 17 proteins were included in the analysis. For the protein‐only panel, multivariable linear regression with stepwise regression reduction was conducted using fasting blood glucose as the response variable and baseline protein levels as the predictor variables. For the integrated panel, multivariable linear regression with stepwise reduction was performed using fasting blood glucose as the response variable and baseline protein levels alongside clinical markers as predictors. The clinical markers (*n* = 14) included in the analysis were commonly available cardiometabolic health indicators: fasting blood glucose, HbA1c, total cholesterol, triglycerides, ALAT, ASAT, CRP, insulin, C‐peptide, calcium, and OGTT blood glucose levels at 30, 60, 90, and 120 min. For both models, elimination was bidirectional, and the Akaike Information Criterion (AIC) was used as the stopping criterion. F‐statistic, *p*‐value, R‐squared, and adjusted R‐squared were extracted for the panels. Estimates (coefficients), standard error, t‐value, *p*‐value, and R‐squared were extracted for each predictor variable. All analyses were performed in R (v 4.4.1), and all plots were generated using the ggplot2 package (v 3.5.1). Protein names, genes, biological processes, and molecular functions were inferred from the Gene Ontology (GO) project. The Q‐Q plot confirmed the normality of the model.

## Author Contributions

Design of the PREVIEW trial by Fogelholm, Raben, and Brand‐Miller. Conceptualization and experimental design: Maria Lastra Cagigas, Andrius Masedunskas, Luigi Fontana. Recruitment, intervention, data collection, and sample collection/biobanking of the Sydney cohort of the PREVIEW Study: Roslyn Muirhead, Jennie Brand‐Miller. Mass spectrometry experiments: Samantha J. Emery‐Corbin, Jumana M. Yousef, Laura F. Dagley. Olink assay: Sam Olechnowicz, Rory Bowden. SASP curation and analysis: Yao Lin, Marco Demaria. Predictive biomarker panel: Rachael Hayward, Gary Low, Maria Lastra Cagigas. Data analysis, interpretation, figures, and manuscript draft: Maria Lastra Cagigas. Revision of data, findings, figures, and text: Andrius Masedunskas, Samantha J. Emery‐Corbin, Sam Olechnowicz, Marco Demaria, Yao Lin, Jennie Brand‐Miller, Stephen J. Fuller, Luigi Fontana. Supervision and funding acquisition: Luigi Fontana, Stephen J. Fuller. All authors revised the final version of the manuscript.

## Conflicts of Interest

L.F. and J.B.M. are authors of widely recognized nutrition books on metabolic health. The remaining authors declare no conflicts of interest. This article was not commissioned, and the funding sources had no role in the study design, data interpretation, or the generation of the findings. No author received financial or other benefits for their contributions to this work.

## Supporting information


**Figure S1.** Related to the LC–MS/MS “neat plasma” and “cEV‐enriched plasma” datasets. Intensities, number of peptides detected, and number of proteins inferred in each method are shown.
**Figure S2.** Related to the LC–MS/MS “neat plasma” and “cEV‐enriched plasma” datasets. Analysis workflow, overlap between the 2 datasets, and direction of the results.
**Figure S3.** Related to the LC–MS/MS “cEV‐enriched plasma” dataset. FunRich results for predicted protein location.
**Figure S4.** Related to the LC–MS/MS “cEV‐enriched plasma” dataset.
**Figure S5.** Full list of IPA of enriched canonical pathways from differentially expressed cEV proteins. Input = cEV dataset.
**Figure S6.** Related to the LC–MS/MS “cEV‐enriched plasma” dataset. Gene Ontology and Reactome enrichment analyses.
**Figure S7.** Related to the LC–MS/MS “neat plasma” dataset. Gene Ontology and Reactome enrichment analyses.
**Figure S8.** Related to the LC–MS/MS “neat plasma” dataset. (A) Proteins significantly correlated with BMI at baseline and were differentially expressed at week 8 relative to baseline. (B) Proteins significantly correlated with fat percentage at baseline and were differentially expressed at week 8 relative to baseline.
**Figure S9.** Related to the Olink dataset.
**Figure S10.** Volcano plot of Olink results with curated SASP list overlapped.
**Figure S11.** Individual abundances for selected proteins from LC–MS/MS and Table 1, paired. Each line represents the protein abundance trajectory in each study participant from baseline to week 8.
**Table S1.** Comparison of baseline anthropometric and cardiometabolic characteristics of the PREVIEW Sydney cohort and selected sub‐cohort. Data expressed as mean ± SD. Statistical significance was calculated using the Wilcoxon rank‐sum test. Significance levels are indicated as *p*‐values.
**Table S2.** Full list of proteins included in the Olink Target Inflammation Panel, as provided by the manufacturer (*n* = 92 proteins).
**Table S3.** Curated SASP protein list, as described in the text (*n* = 163 proteins).
**Table S4.** Predictor panels for fasting glucose response.

## Data Availability

A comprehensive description of the trial design, study population, interventions, and outcomes is available in previous publications of the PREVIEW study (ClinicalTrials.gov identifier: NCT01777893). The mass spectrometry‐based proteomics data have been deposited in the ProteomeXchange Consortium via the PRIDE partner repository under the dataset identifier PXD056694. A complete list of senescence‐associated proteins is provided in the Supportng Material. Additional data are available from the corresponding author upon reasonable request.
