# Supplementary material for: Short‐Term Severe Energy Restriction Promotes Molecular Health and Reverses Aging Signatures in Adults With Prediabetes in the PREVIEW Study
Source: Aging Cell. 2025 Jun 16;24(8):e70123. doi: 10.1111/acel.70123 (PMC12341815; doi:10.1111/acel.70123)
Supplement: Supplementary file 1 — Figure S1. Related to the LC–MS/MS “neat plasma” and “cEV‐enriched plasma” datasets. Intensities, number of peptides detected, and number of proteins inferred in each method are shown. Figure S2. Related to the LC–MS/MS “neat plasma” and “cEV‐enriched plasma” datasets. Analysis workflow, overlap between the 2 datasets, and direction of the results. Figure S3. Related to the LC–MS/MS “cEV‐enriched plasma” dataset. FunRich results for predicted protein location. Figure S4. Related to the LC–MS/MS “cEV‐enriched plasma” dataset. Figure S5. Full list of IPA of enriched canonical pathways from differentially expressed cEV proteins. Input = cEV dataset. Figure S6. Related to the LC–MS/MS “cEV‐enriched plasma” dataset. Gene Ontology and Reactome enrichment analyses. Figure S7. Related to the LC–MS/MS “neat plasma” dataset. Gene Ontology and Reactome enrichment analyses. Figure S8. Related to the LC–MS/MS “neat plasma” dataset. (A) Proteins significantly correlated with BMI at baseline and were differentially expressed at week 8 relative to baseline. (B) Proteins significantly correlated with fat percentage at baseline and were differentially expressed at week 8 relative to baseline. Figure S9. Related to the Olink dataset. Figure S10. Volcano plot of Olink results with curated SASP list overlapped. Figure S11. Individual abundances for selected proteins from LC–MS/MS and Table 1, paired. Each line represents the protein abundance trajectory in each study participant from baseline to week 8. Table S1. Comparison of baseline anthropometric and cardiometabolic characteristics of the PREVIEW Sydney cohort and selected sub‐cohort. Data expressed as mean ± SD. Statistical significance was calculated using the Wilcoxon rank‐sum test. Significance levels are indicated as p‐values. Table S2. Full list of proteins included in the Olink Target Inflammation Panel, as provided by the manufacturer (n = 92 proteins). Table S3. Curated SASP protein list, as described in the text (n = 163 p [file ACEL-24-e70123-s001.pdf]

Supplementary Material

Variable at baseline	Sydney cohort (N=151)	Selected sub-cohort (N=44)	p value
Sex, Female (%)	105 (69.5%)	19 (43.2%)	0.001
Age (years)	53.4 (10.3)	53.7 (9.5)	0.994
Height (m)	167.5 (8.6)	171.9 (8.0)	0.001
Weight (kg)	100.6 (20.6)	102.1 (14.5)	0.296
BMI (kg/m²)	35.8 (6.7)	34.6 (4.9)	0.513
Waist circumference (cm)	114.6 (12.7)	117.0 (11.1)	0.199
Hip circumference (cm)	122.0 (15.1)	118.1 (11.7)	0.182
Fat mass (kg)	43.0 (13.3)	40.2 (9.7)	0.329
Fat-free mass (kg)	55.9 (10.8)	60.3 (9.4)	0.007
Body fat (%)	43.1 (6.8)	39.7 (6.6)	0.006
Fasting plasma glucose (mmol/L)	6.2 (0.6)	6.4 (0.6)	0.184
2-h plasma glucose (mmol/L)	8.6 (2.3)	8.4 (2.1)	0.664
Fasting insulin (mU/L)	13.1 (6.9)	13.9 (6.5)	0.362
C-peptide (pmol/L)	994.2 (346.0)	1035.9 (329.2)	0.400
HOMA-IR	3.7 (2.0)	3.9 (1.8)	0.235
HbA1c (%)	5.7 (0.4)	5.7 (0.4)	0.863
HbA1c (mmol/mol)	38.7 (4.1)	38.6 (4.0)	0.815
Total cholesterol (mmol/L)	4.9 (0.9)	5.0 (0.8)	0.475
HDL cholesterol (mmol/L)	1.2 (0.2)	1.2 (0.2)	0.649
LDL cholesterol (mmol/L)	3.0 (0.8)	3.1 (0.7)	0.698
Triglycerides (mmol/L)	1.6 (0.9)	1.7 (0.9)	0.169
Systolic blood pressure (mmHg)	124.3 (14.6)	130.2 (14.0)	0.013
Diastolic blood pressure (mmHg)	79.5 (9.6)	82.1 (10.4)	0.088
hs-CRP (mg/L)	5.9 (6.4)	4.7 (5.2)	0.344
Weight loss Post-CR (kg)	11.0 (3.5)	14.0 (2.7)	< 0.001
Weight loss Post-CR (%)	10.9 (2.6)	13.7 (1.6)	< 0.001

Supp Table 1

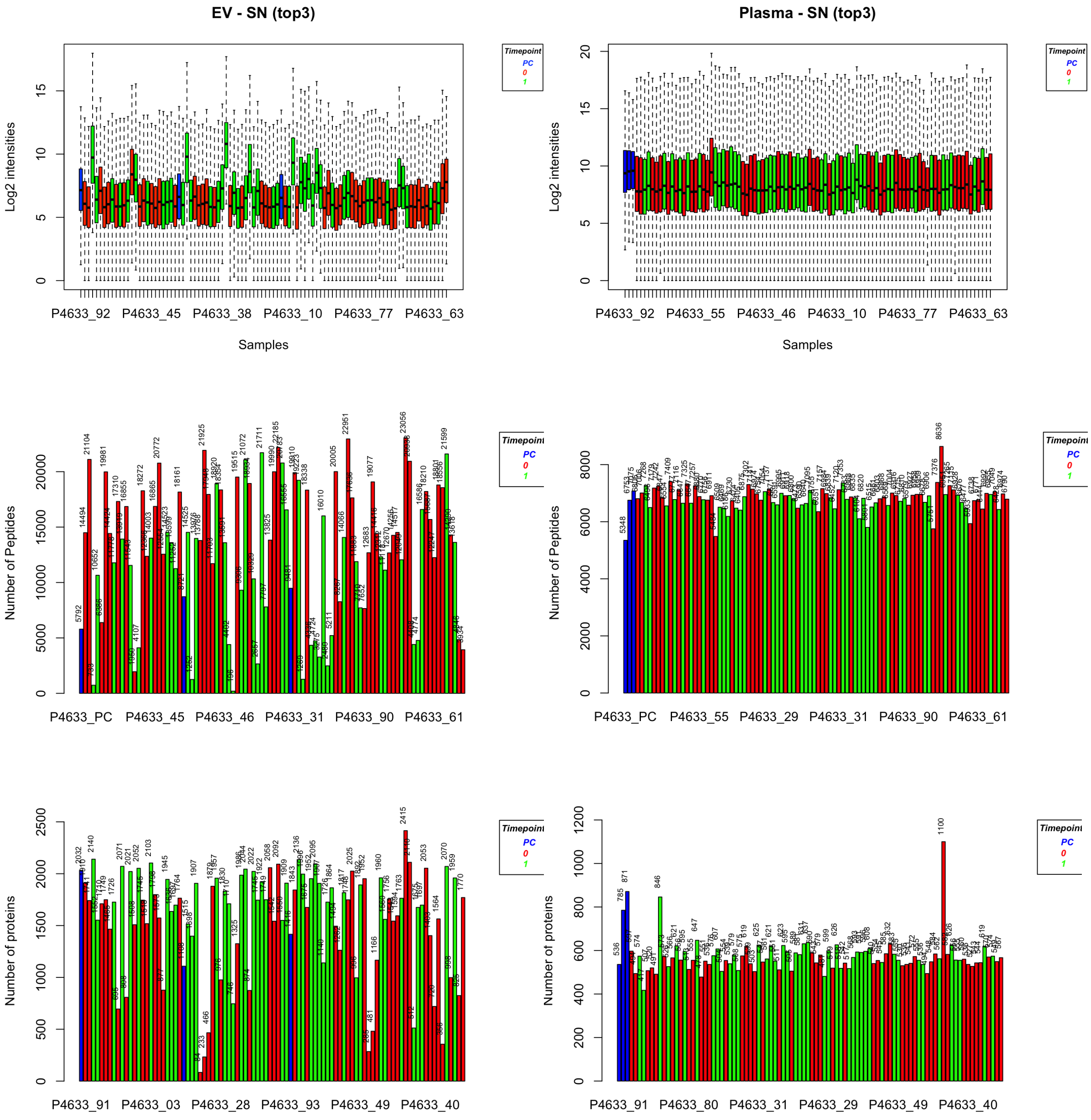

Supp Figure 1

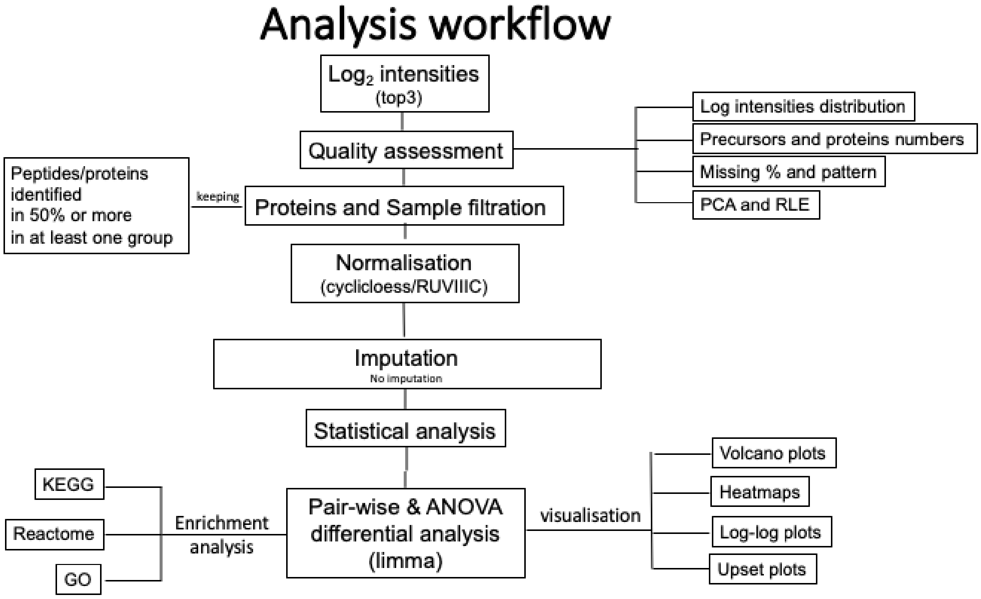

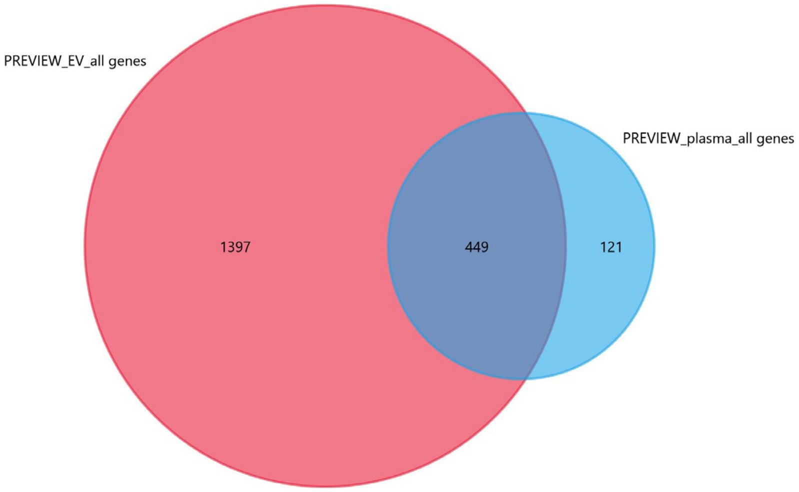

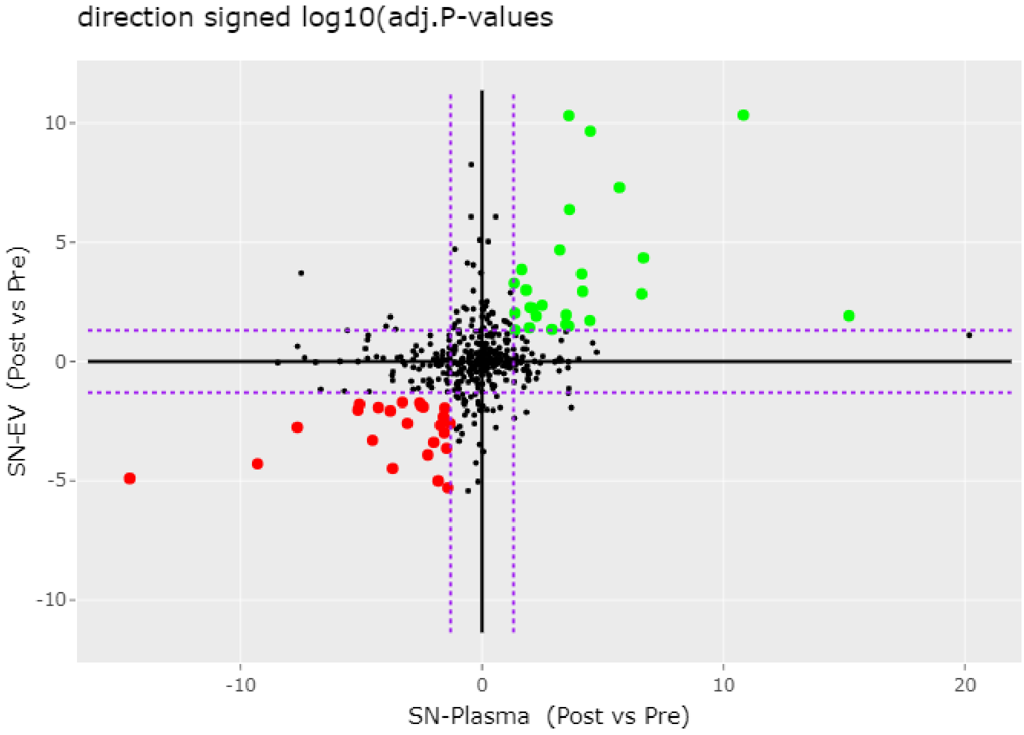

Supp Figure 2

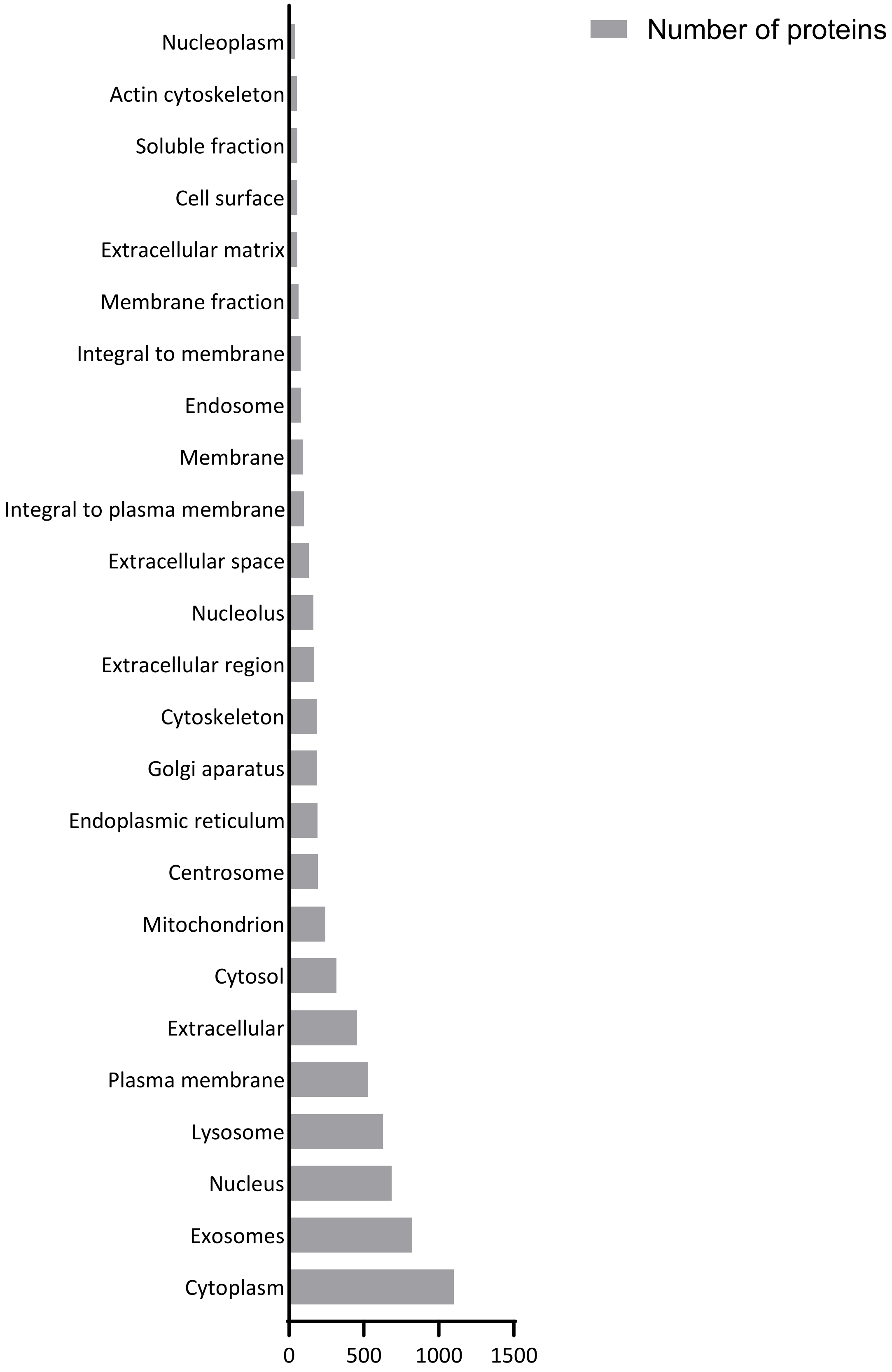

Supp Figure 3

Rank	Vesicle-associated protein (Vesiclepedia)	Vesicle-enriched plasma dataset	Log(FC)	P-value	Adjusted P-value	Significance
1	CD63	CD63	-0.658	1.6E-05	3.3E-04	Down
2	CD9	CD9	-0.360	2.7E-04	2.8E-03	Down
3	PDCD6IP	PDCD6IP	-0.153	7.2E-02	1.8E-01	NotSig
4	TSG101	TSG101	0.083	5.5E-01	7.1E-01	NotSig
5	CD81	CD81	-0.054	6.9E-01	8.1E-01	NotSig
6	GAPDH	GAPDH	0.156	7.8E-02	1.8E-01	NotSig
7	FLOT1	FLOT1	-0.659	1.1E-06	4.1E-05	Down
8	ACTB	ACTB	-0.079	8.3E-01	9.0E-01	NotSig
9	ANXA2	ANXA2	0.003	9.8E-01	9.9E-01	NotSig
10	SDCBP	SDCBP	-0.790	4.4E-06	1.3E-04	Down
11	HSP90AA1	HSP90AA1	-0.280	2.6E-03	1.6E-02	Down
12	HSPA8	HSPA8	-0.266	1.1E-03	8.5E-03	Down
13	ANXA5	ANXA5	-0.212	5.6E-02	1.5E-01	NotSig
14	ENO1	ENO1	0.095	2.0E-01	3.7E-01	NotSig
15	PKM	PKM	-0.037	5.4E-01	7.0E-01	NotSig
16	HSP90AB1	HSP90AB1	-0.276	5.6E-03	2.8E-02	Down
17	YWHAZ	YWHAZ	-0.072	2.9E-01	4.7E-01	NotSig
18	PGK1	PGK1	-0.164	7.3E-02	1.8E-01	NotSig
19	YWHAE	YWHAE	-0.096	1.5E-01	3.1E-01	NotSig
20	FLOT2	FLOT2	-0.467	9.2E-04	7.5E-03	Down
21	ANXA1	ANXA1	0.254	1.2E-01	2.5E-01	NotSig
22	VCP	VCP	-0.178	3.7E-02	1.1E-01	NotSig
23	PPIA	PPIA	-0.102	1.5E-01	3.1E-01	NotSig
24	EEF1A1	Missing				
25	ALB	ALB	0.097	1.5E-01	3.0E-01	NotSig
26	ALDOA	ALDOA	-0.010	8.7E-01	9.2E-01	NotSig
27	ITGB1	ITGB1	0.106	9.7E-02	2.2E-01	NotSig
28	MYH9	MYH9	-0.096	5.5E-01	7.1E-01	NotSig
29	CLTC	CLTC	-0.688	1.5E-04	1.8E-03	Down
30	TPI1	TPI1	-0.106	1.7E-01	3.3E-01	NotSig
31	CFL1	CFL1	-0.042	6.5E-01	7.9E-01	NotSig
32	EEF2	EEF2	0.067	7.3E-01	8.4E-01	NotSig
33	MSN	MSN	-0.077	2.7E-01	4.5E-01	NotSig
34	GNB1	GNB1	-0.127	9.2E-02	2.1E-01	NotSig
35	PRDX1	PRDX1	0.021	7.8E-01	8.7E-01	NotSig
36	ANXA6	ANXA6	-0.055	6.5E-01	7.9E-01	NotSig
37	SLC3A2	SLC3A2	-0.759	9.4E-04	7.5E-03	Down
38	EZR	EZR	-0.433	5.8E-02	1.5E-01	NotSig
39	LDHA	LDHA	-0.255	5.0E-03	2.6E-02	Down
40	LDHB	LDHB	-0.203	5.0E-02	1.4E-01	NotSig
41	Bsg	Bsg	0.020	6.9E-01	8.2E-01	NotSig
42	Cdc42	Cdc42	-0.187	3.2E-03	1.8E-02	Down
43	PFN1	PFN1	-0.163	1.8E-01	3.4E-01	NotSig
44	ATP1A1	ATP1A1	-0.170	1.2E-01	2.6E-01	NotSig
45	ACTN4	ACTN4	-0.225	3.2E-01	5.0E-01	NotSig
46	HSPA1A	Missing				
47	FLNA	FLNA	-0.239	3.5E-02	1.1E-01	NotSig
48	YWHAB	YWHAB	-0.188	6.4E-02	1.6E-01	NotSig
49	GNAI2	GNAI2	-0.195	4.8E-03	2.5E-02	Down
50	YWHAQ	YWHAQ	-0.035	6.2E-01	7.7E-01	NotSig
51	FASN	FASN	0.147	4.5E-01	6.3E-01	NotSig
52	CLIC1	CLIC1	-0.051	3.7E-01	5.5E-01	NotSig
53	PRDX2	PRDX2	-0.697	2.3E-04	2.5E-03	Down
54	GSN	GSN	0.626	3.9E-07	1.9E-05	Up
55	CCT2	CCT2	-0.153	2.9E-01	4.7E-01	NotSig
56	RAB5C	RAB5C	-0.173	1.0E-01	2.3E-01	NotSig
57	HIST1H4A	H4C1 HIST1H4A	0.045	8.6E-01	9.2E-01	NotSig
58	RAP1B	RAP1B	0.427	1.8E-01	3.5E-01	NotSig
59	GNB2	GNB2	0.078	2.4E-01	4.2E-01	NotSig
60	LGALS3BP	LGALS3BP	-0.693	8.0E-07	3.2E-05	Down
61	YWHAG	YWHAG	-0.143	1.1E-01	2.5E-01	NotSig
62	RAB10	RAB10	-0.083	3.7E-01	5.6E-01	NotSig
63	HLA-A	HLA-A	-0.396	1.2E-03	9.0E-03	Down
64	ACTN1	ACTN1	0.083	5.7E-01	7.3E-01	NotSig
65	ANXA7	ANXA7	-0.600	2.3E-04	2.5E-03	Down
66	FN1	FN1	-0.102	4.0E-01	5.8E-01	NotSig
67	TFRC	TFRC	-0.625	1.3E-04	1.7E-03	Down
68	RAN	RAN	-0.291	1.7E-02	6.5E-02	NotSig
69	GDI2	GDI2	-0.040	5.8E-01	7.3E-01	NotSig
70	CCT3	CCT3	-0.107	3.3E-01	5.1E-01	NotSig
71	AHCY	AHCY	-0.633	2.5E-04	2.7E-03	Down
72	HSPA5	HSPA5	-0.206	2.4E-02	8.0E-02	NotSig
73	CCT4	CCT4	-0.238	1.4E-02	5.4E-02	NotSig
74	ACLY	ACLY	-0.235	5.2E-02	1.4E-01	NotSig
75	C3	C3	0.026	7.8E-01	8.7E-01	NotSig
76	UBA1	UBA1	0.055	7.4E-01	8.5E-01	NotSig
77	ANXA11	ANXA11	-0.137	1.8E-01	3.5E-01	NotSig
78	TUBB4B	TUBB4B	-0.070	5.4E-01	7.1E-01	NotSig
79	KPNB1	KPNB1	-0.281	4.0E-02	1.2E-01	NotSig
80	CAP1	CAP1	-0.243	7.4E-02	1.8E-01	NotSig
81	Rac1	Rac1	-0.067	5.3E-01	7.0E-01	NotSig
82	MFGE8	MFGE8	-0.575	2.7E-04	2.8E-03	Down
83	TCP1	TCP1	-0.239	1.4E-02	5.4E-02	NotSig
84	RHOA	RHOA	-0.193	4.2E-01	6.0E-01	NotSig
85	TLN1	TLN1	-0.002	9.8E-01	9.9E-01	NotSig
86	CCT6A	CCT6A	-0.425	1.3E-03	9.5E-03	Down
87	GNAS	Missing				
88	Cct5	Cct5	-0.475	5.2E-02	1.4E-01	NotSig
89	Rala	Rala	0.039	8.1E-01	8.9E-01	NotSig
90	EHD1	EHD1	-0.079	3.8E-01	5.6E-01	NotSig
91	CCT8	CCT8	-0.072	4.0E-01	5.8E-01	NotSig
92	PGAM1	PGAM1	-0.091	3.8E-01	5.6E-01	NotSig
93	IQGAP1	IQGAP1	-0.276	4.2E-01	6.0E-01	NotSig
94	VCL	VCL	-0.092	2.7E-01	4.4E-01	NotSig
95	GPI	GPI	-0.103	7.2E-02	1.7E-01	NotSig
96	EIF4A1	EIF4A1	-0.029	7.5E-01	8.5E-01	NotSig
97	RAB7A	RAB7A	0.102	2.0E-01	3.7E-01	NotSig
98	EEF1G	EEF1G	-0.054	6.7E-01	8.0E-01	NotSig
99	ADAM10	ADAM10	-0.133	8.3E-02	1.9E-01	NotSig
100	A2M	A2M	0.228	1.5E-01	3.0E-01	NotSig

Supp Figure 4

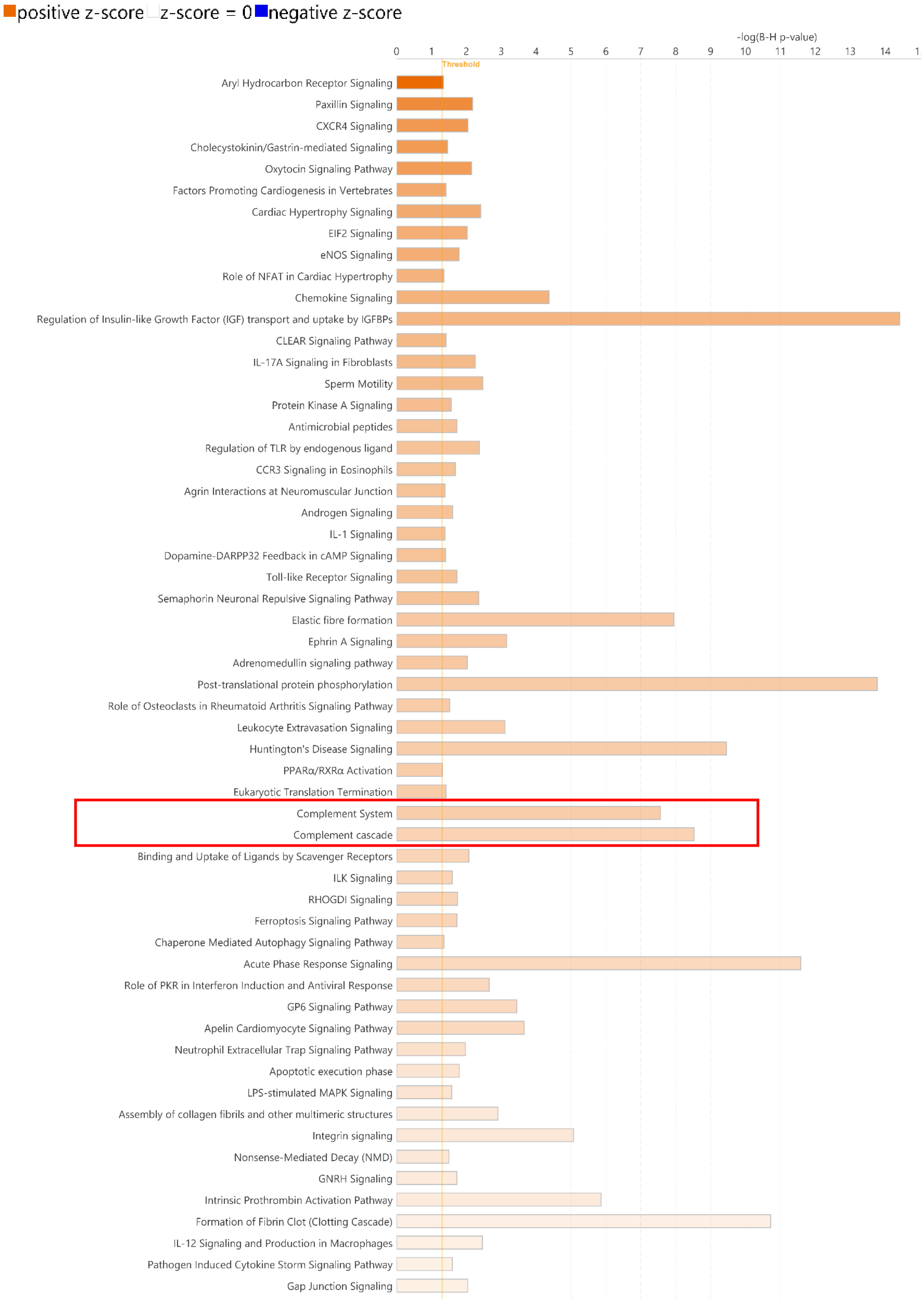

Supp Figure 5

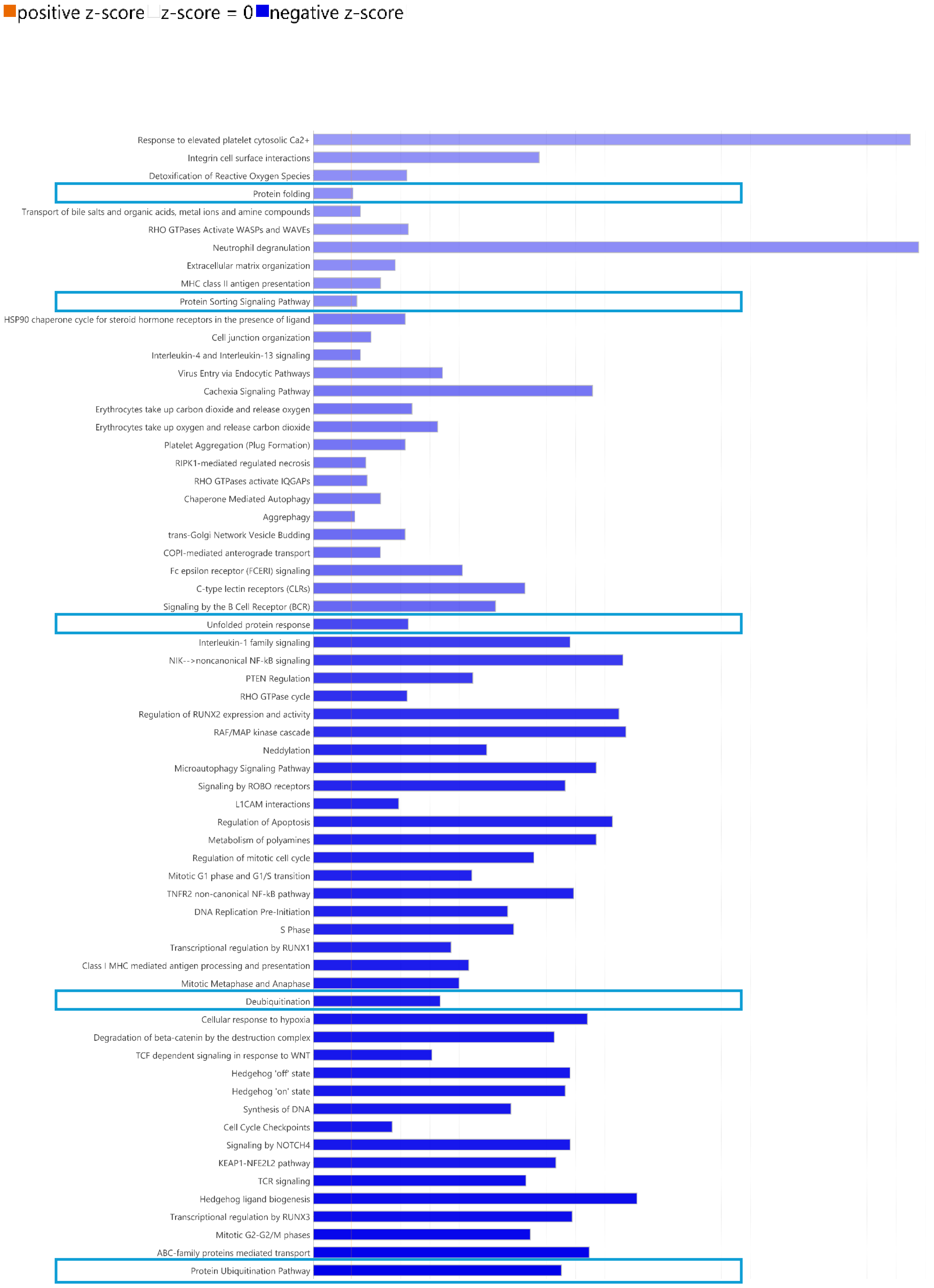

Supp Figure 5 (continued)

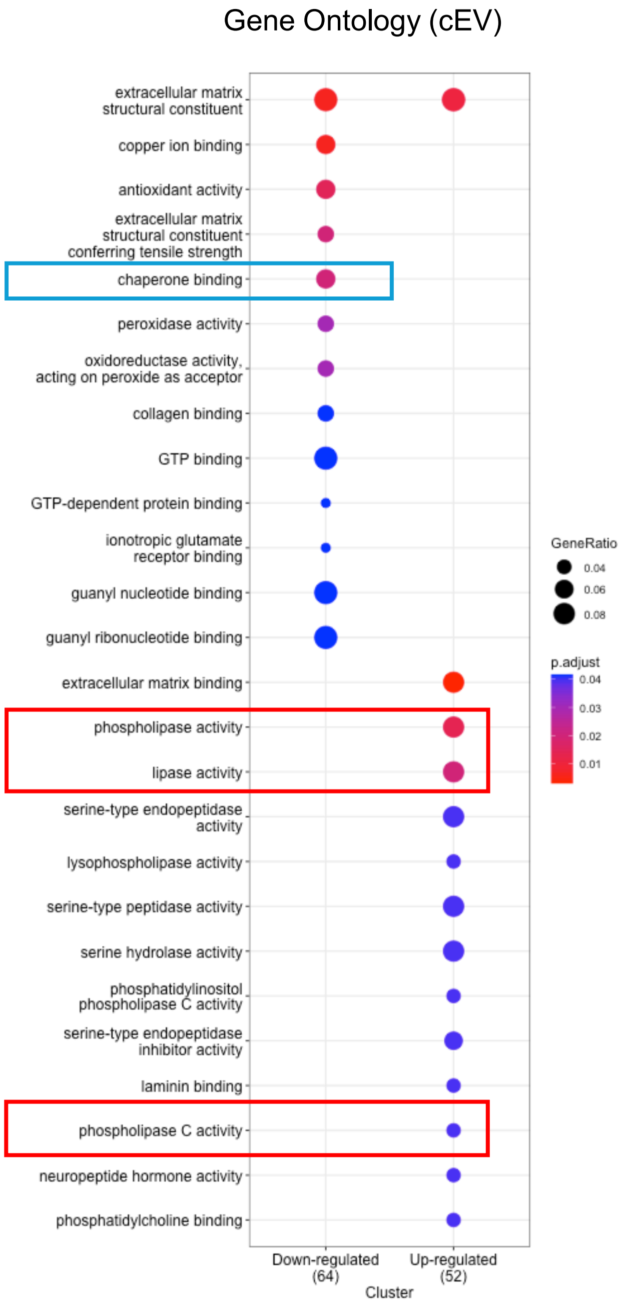

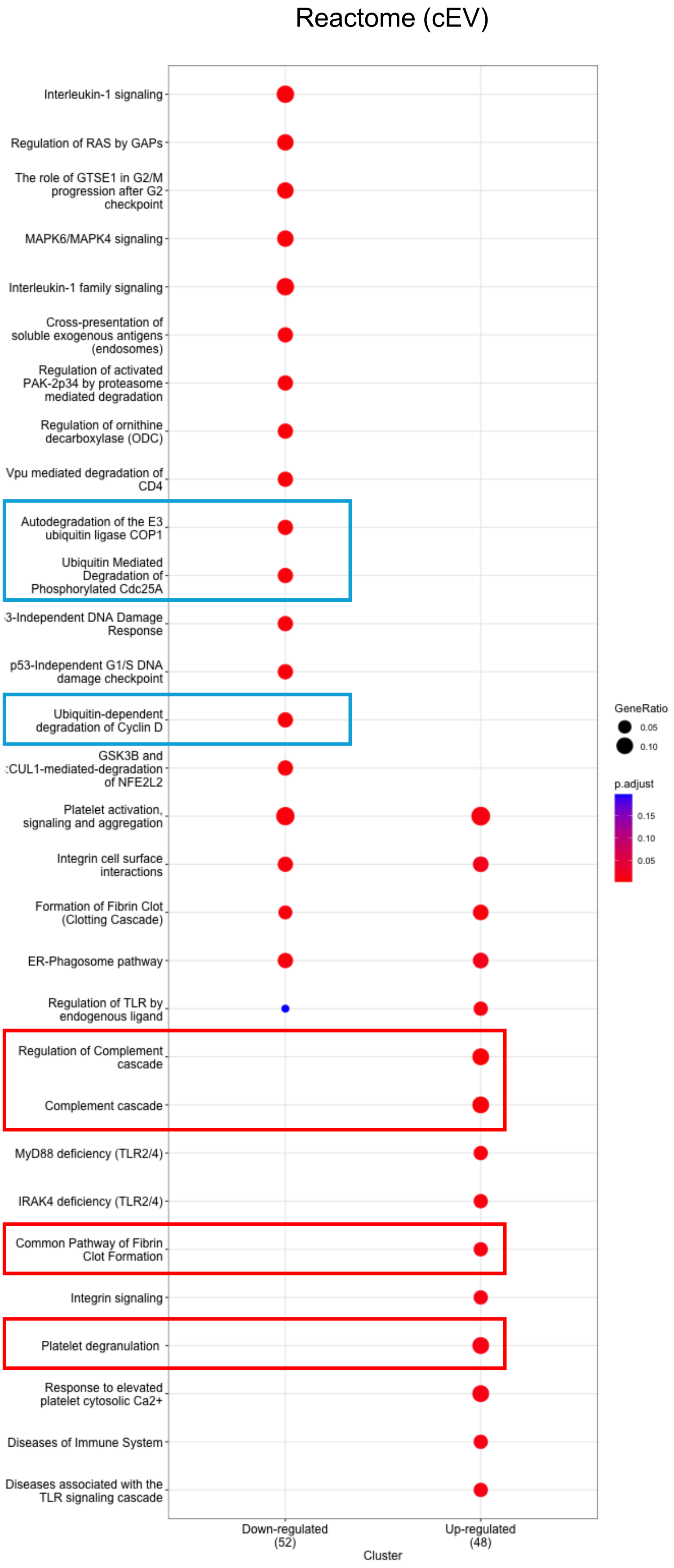

Supp Figure 6

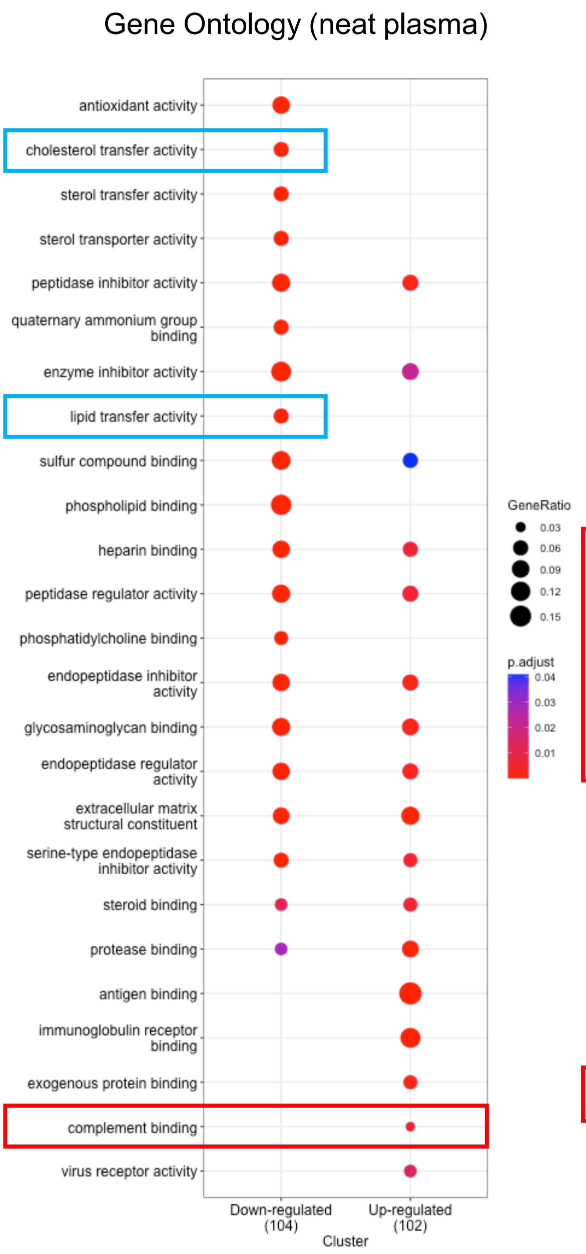

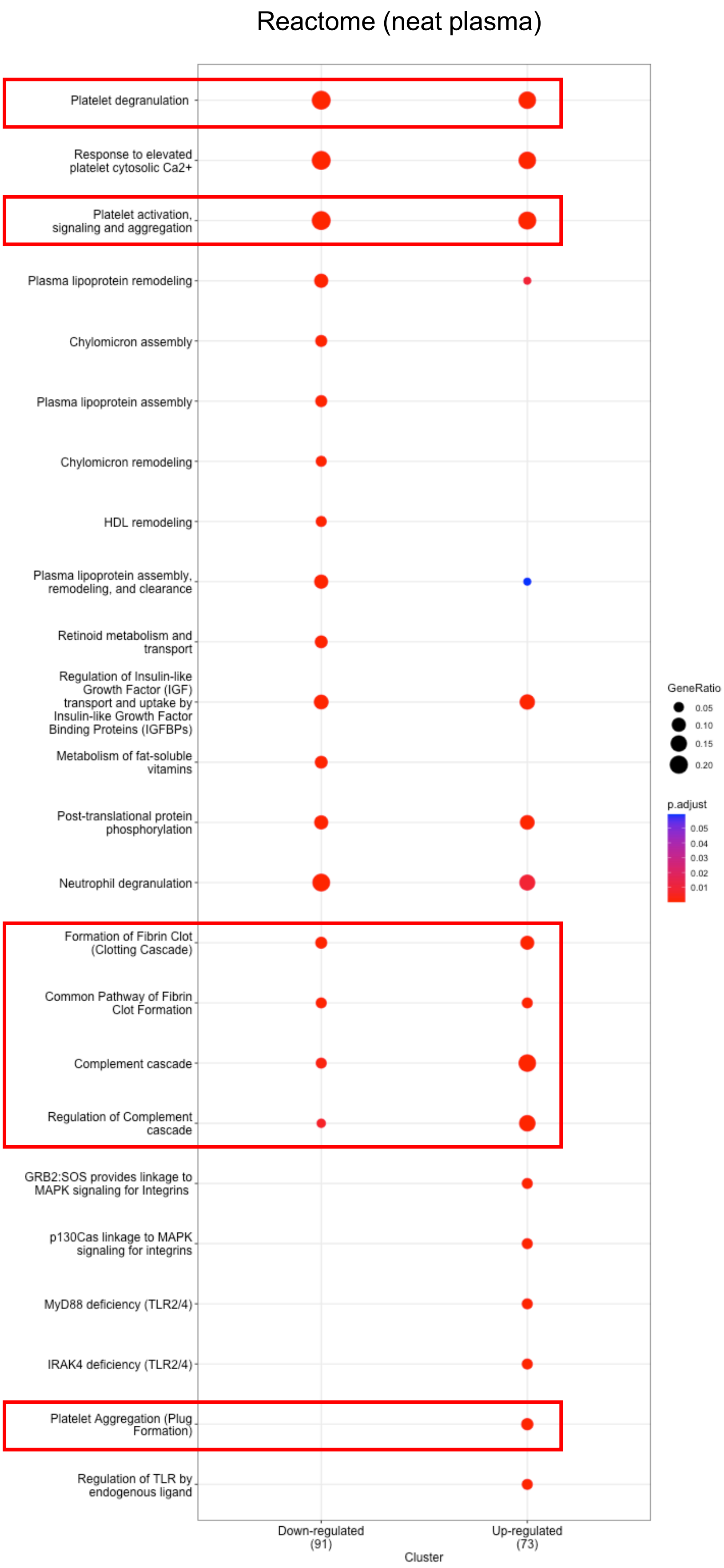

Supp Figure 7

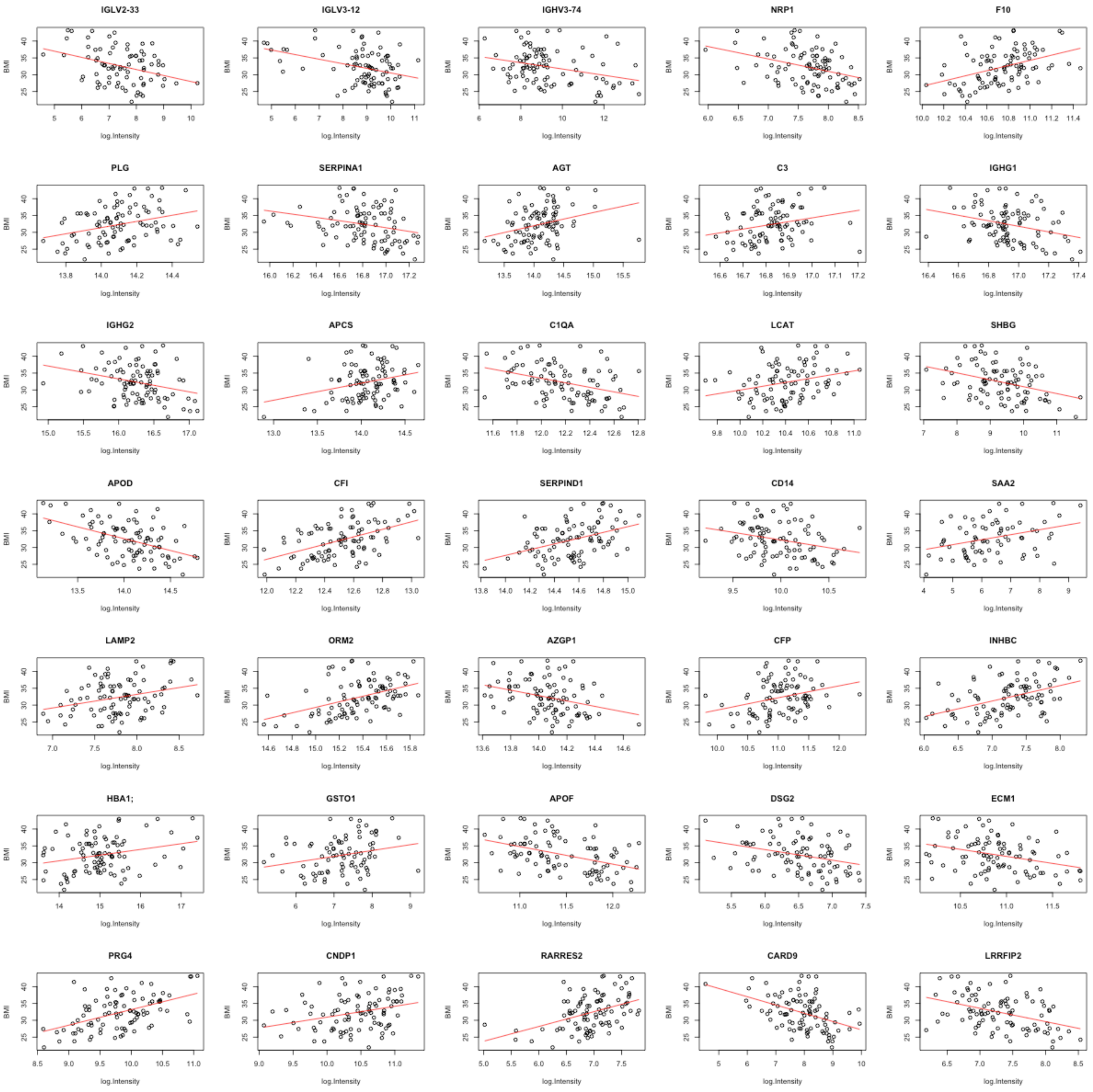

Supp Figure 8A

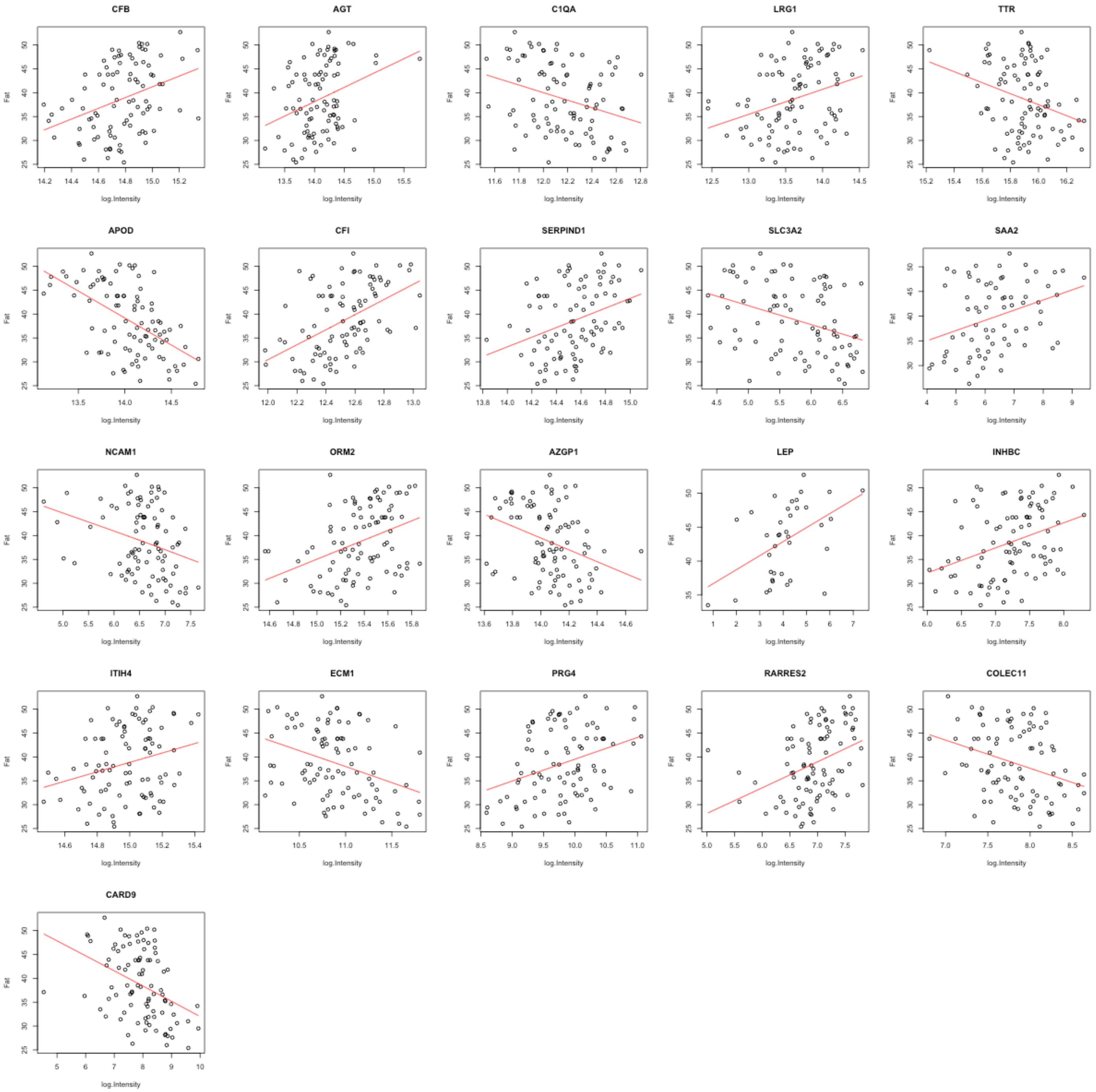

Supp Figure 8B

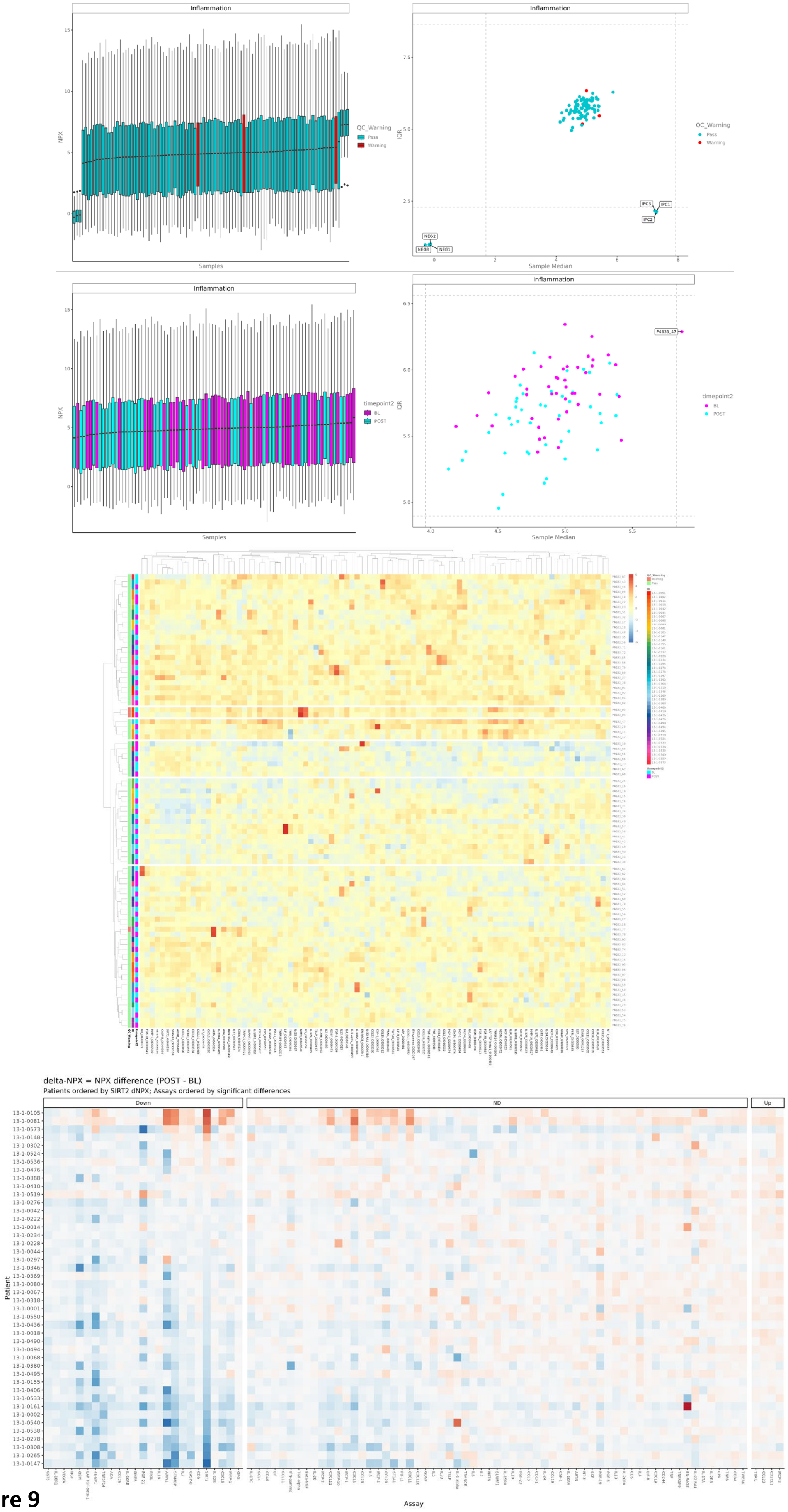

Supp Figure 9

Olink Assay	Uniprot ID	OlinkID
IL8	P10145	OID00471
VEGFA	P15692	OID00472
CD8A	P01732	OID05124
MCP-3	P80098	OID00474
GDNF	P39905	OID00475
CDCP1	Q9H5V8	OID00476
CD244	Q9BZW8	OID00477
IL7	P13232	OID00478
OPG	O00300	OID00479
LAP TGF-beta-1	P01137	OID00480
uPA	P00749	OID00481
IL6	P05231	OID00482
IL-17C	Q9P0M4	OID00483
MCP-1	P13500	OID00484
IL-17A	Q16552	OID00485
CXCL11	O14625	OID00486
AXIN1	O15169	OID00487
TRAIL	P50591	OID00488
IL-20RA	Q9UHF4	OID00489
CXCL9	Q07325	OID00490
CST5	P28325	OID00491
IL-2RB	P14784	OID00492
IL-1 alpha	P01583	OID00493
OSM	P13725	OID00494
IL2	P60568	OID00495
CXCL1	P09341	OID00496
TSLP	Q969D9	OID00497
CCL4	P13236	OID00498
CD6	P30203	OID00499
SCF	P21583	OID00500
IL18	Q14116	OID00501
SLAMF1	Q13291	OID00502
TGF-alpha	P01135	OID00503
MCP-4	Q99616	OID00504
CCL11	P51671	OID00505
TNFSF14	O43557	OID00506
FGF-23	Q9GZV9	OID00507
IL-10RA	Q13651	OID00508
FGF-5	P12034	OID00509
MMP-1	P03956	OID00510
LIF-R	P42702	OID00511
FGF-21	Q9NSA1	OID00512
CCL19	Q99731	OID00513
IL-15RA	Q13261	OID00514
IL-10RB	Q08334	OID00515
IL-22 RA1	Q8N6P7	OID00516
IL-18R1	Q13478	OID00517
PD-L1	Q9NZQ7	OID00518
Beta-NGF	P01138	OID00519
CXCL5	P42830	OID00520
TRANCE	O14788	OID00521
HGF	P14210	OID00522
IL-12B	P29460	OID00523
IL-24	Q13007	OID00524
IL13	P35225	OID00525
ARTN	Q5T4W7	OID00526
MMP-10	P09238	OID00527
IL10	P22301	OID00528
TNF	P01375	OID05548
CCL23	P55773	OID00530
CD5	P06127	OID00531
CCL3	P10147	OID00532
Flt3L	P49771	OID00533
CXCL6	P80162	OID00534
CXCL10	P02778	OID00535
4E-BP1	Q13541	OID00536
IL-20	Q9NYY1	OID00537
SIRT2	Q8IXJ6	OID00538
CCL28	Q9NRJ3	OID00539
DNER	Q8NFT8	OID01213
EN-RAGE	P80511	OID00541
CD40	P25942	OID00542
IL33	O95760	OID00543
IFN-gamma	P01579	OID05547
FGF-19	O95750	OID00545
IL4	P05112	OID00546
LIF	P15018	OID00547
NRTN	Q99748	OID00548
MCP-2	P80075	OID00549
CASP-8	Q14790	OID00550
CCL25	O15444	OID00551
CX3CL1	P78423	OID00552
TNFRSF9	Q07011	OID00553
NT-3	P20783	OID00554
TWEAK	O43508	OID00555
CCL20	P78556	OID00556
ST1A1	P50225	OID00557
STAMBP	O95630	OID00558
IL5	P05113	OID00559
ADA	P00813	OID00560
TNFB	P01374	OID00561
CSF-1	P09603	OID00562

Supp Table 2

Post-intervention vs Baseline

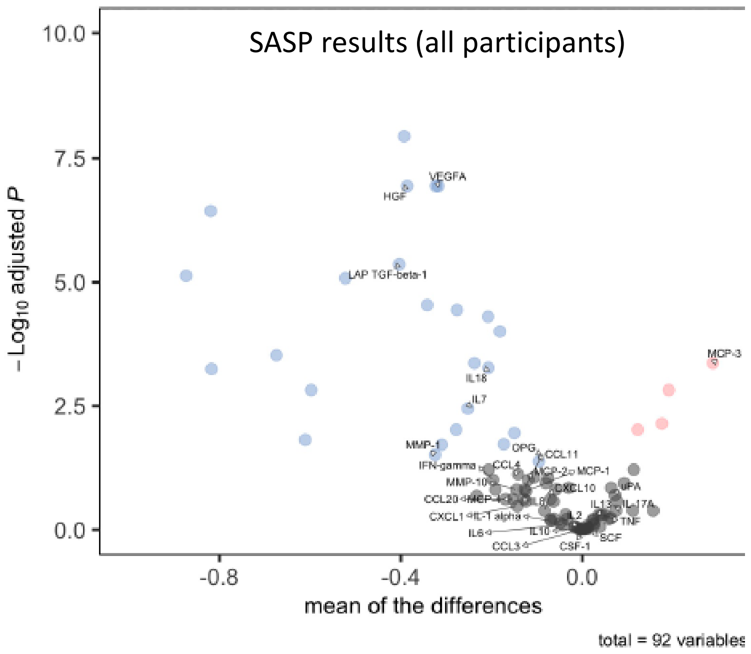

Post-intervention vs Baseline

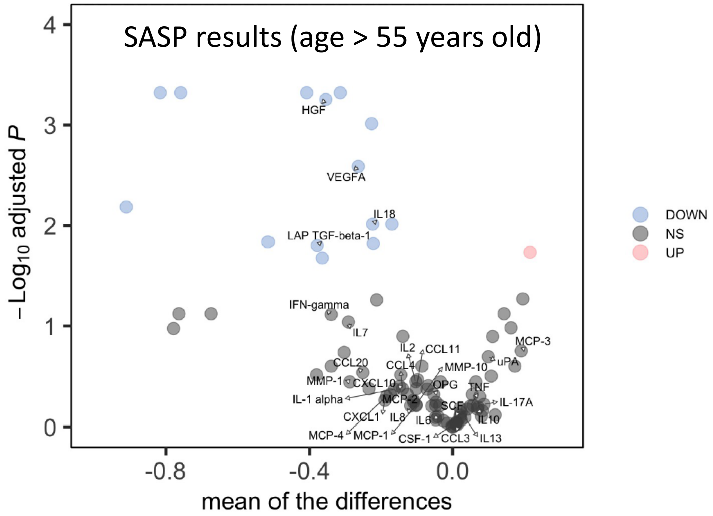

Age-effect results (age > 55 years old)

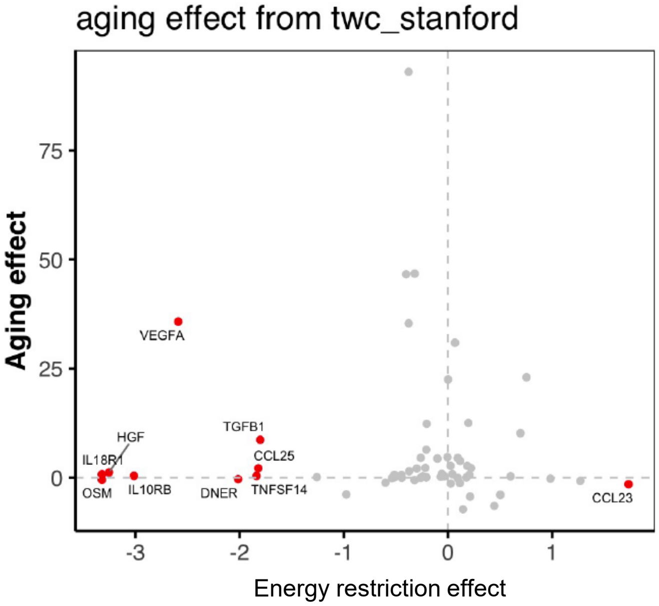

Age-effect results (age > 55 years old)

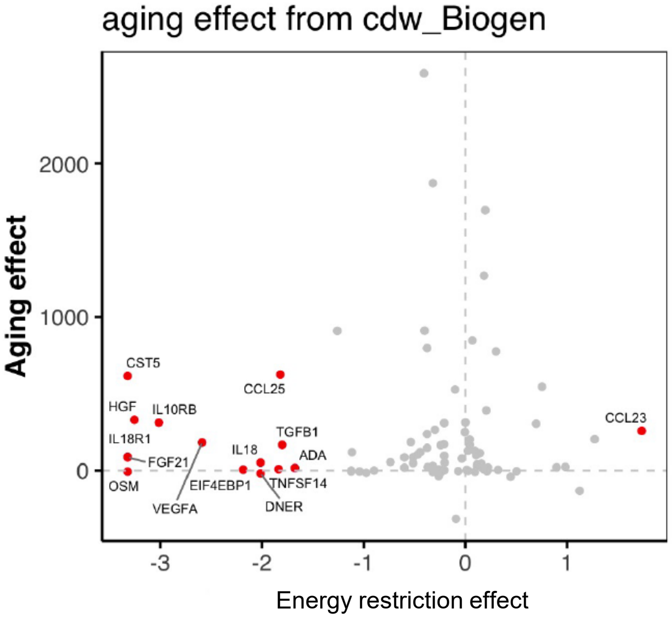

Supp Figure 10

Curated SASP protein list (n = 163 proteins)

ACVR1B	CSF1	GDF15	IL7	PLAUR	ADAMTS13	MPO
ANG	CSF2	GEM	INHA	PTBP1	ALCAM	PARK7
ANGPT1	CSF2RB	GMFG	IQGAP2	PTGER2	BGN	PGK1
ANGPTL4	CST4	HGF	ITGA2	PTGES	CCL11	POSTN
AREG	CTNNB1	HMGB1	ITPKA	RPS6KA5	CCL17	RB1
AXL	CTSB	ICAM1	JUN	SCAMP4	CCL18	SERPING1
BEX3	CXCL1	ICAM3	KITLG	SELPLG	CCL22	SOST
BMP2	CXCL10	IGF1	LCP1	SEMA3F	CLEC11A	STC1
BMP6	CXCL12	IGFBP1	MIF	SERPINB4	COTL1	TGFB1
C3	CXCL16	IGFBP2	MMP1	SERPINE1	CST3	TIMP1
CCL1	CXCL2	IGFBP3	MMP10	SERPINE2	CTSD	TP53
CCL13	CXCL3	IGFBP4	MMP12	SPP1	CTSZ	YWHAQ
CCL16	CXCL8	IGFBP5	MMP13	SPX	FN1	SERPINB2
CCL2	CXCR2	IGFBP6	MMP14	TIMP2	GSN	
CCL20	DKK1	IGFBP7	MMP2	TNF	GSTP1	
CCL24	EDN1	IL10	MMP3	TNFRSF10C	HSPA1A	
CCL26	EGF	IL13	MMP9	TNFRSF11B	HSPA8	
CCL3	EGFR	IL15	NAP1L4	TNFRSF1A	IFNG	
CCL3L1	EREG	IL18	NRG1	TNFRSF1B	IL17A	
CCL4	ESM1	IL1A	PAPPA	TUBGCP2	IL1R1	
CCL5	ETS2	IL1B	PECAM1	VEGFA	IL9	
CCL7	FAS	IL2	PGF	VEGFC	INHBA	
CCL8	FGF1	IL32	PIGF	VGF	LAMB1	
CD55	FGF2	IL6	PLAT	WNT16	MMP7	
CD9	FGF7	IL6ST	PLAU	WNT2	MOK	

Supp Table 3

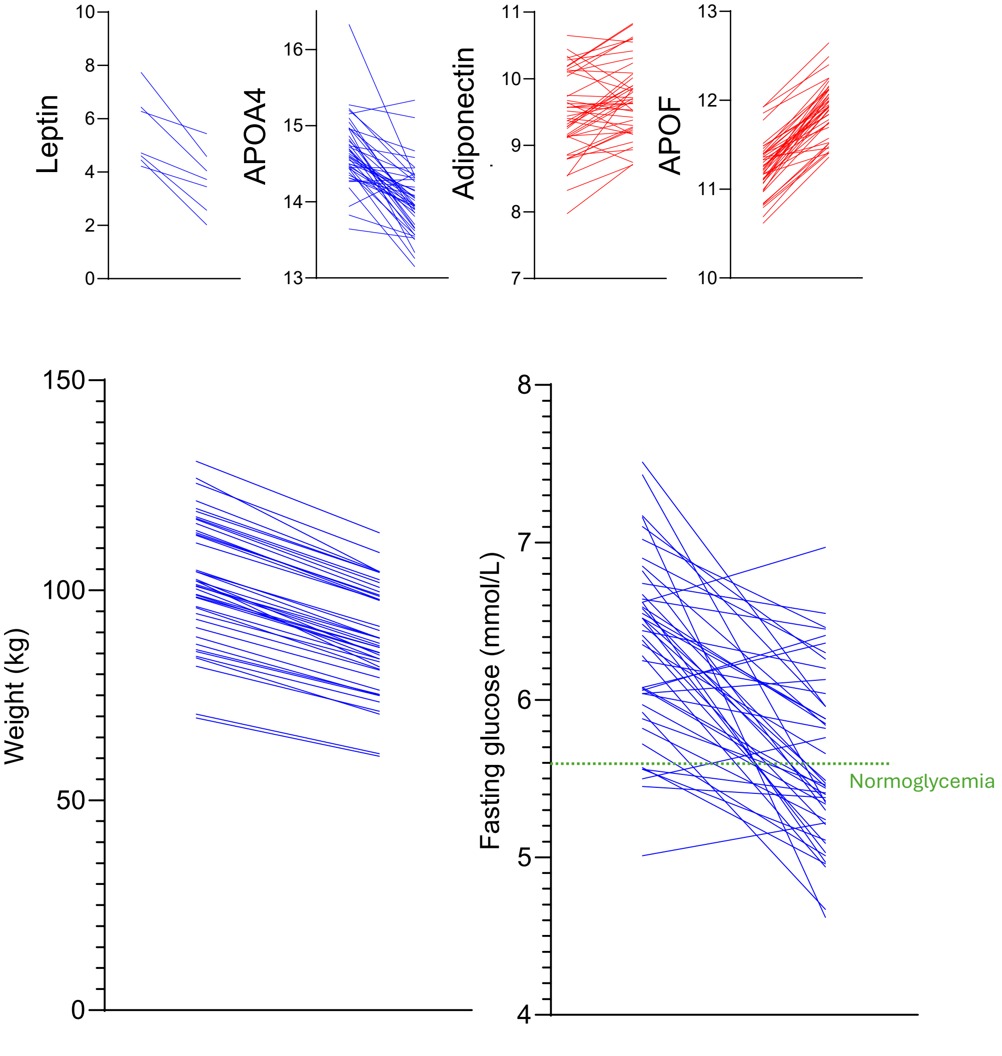

Supp Figure 11

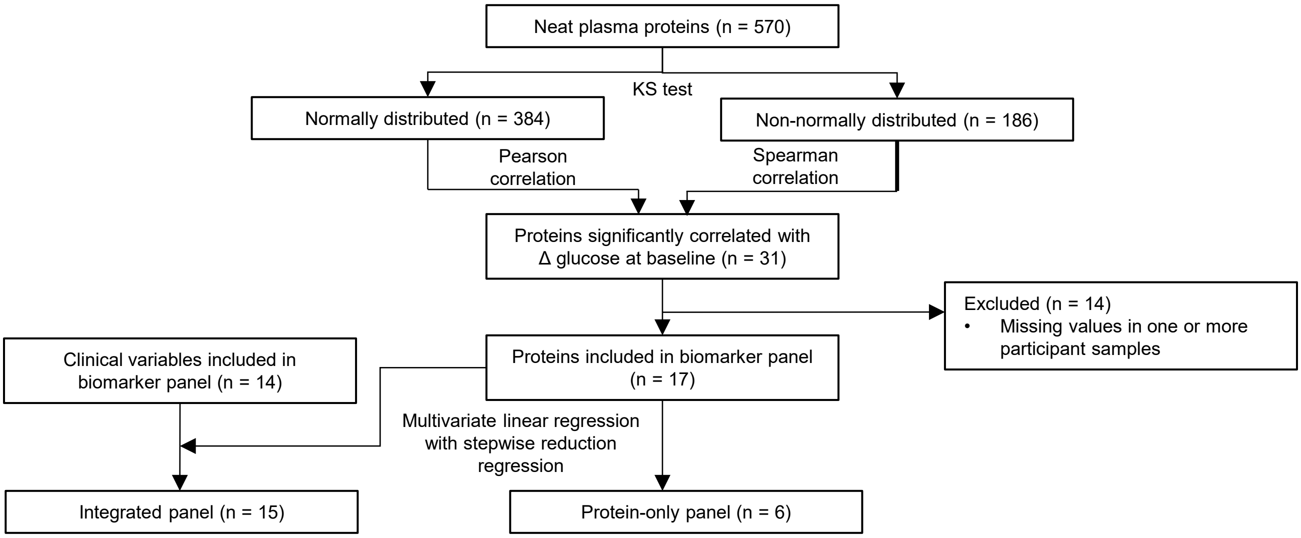

Predictor variable	Estimate	Standard error	P value	F statistic	R^2	Adjusted R^2	Residual standard error
Protein-only panel			0.000207	5.93 (6,37)	49.01%	40.74%	0.503
Mannan-binding lectin serine protease 2	0.573	0.198	0.00626				
Insulin-like growth factor-binding protein complex acid labile subunit	0.948	0.316	0.00483				
Immunoglobulin heavy constant gamma 1	-1.43	0.541	0.0119				
Insulin-like growth factor-binding protein 3	-0.711	0.373	0.0647				
Complement C1q subcomponent subunit A	-0.579	0.384	0.140				
Protein AMBP	0.500	0.358	0.171				
Integrated panel			0.000003 24	7.38 (15,28)	79.83%	69.02%	0.363
Platelet glycoprotein Ib alpha chain	0.243	0.0712	0.00197				
Retinoic acid receptor responder protein 2	0.648	0.226	0.00767				
Inter-alpha-trypsin inhibitor heavy chain H1	0.594	0.3336	0.08588				
Immunoglobulin heavy constant gamma 1	-0.627	0.378	0.108				
Properdin	-0.288	0.187	0.135				
Phosphatidylcholine-sterol acyltransferase	-0.420	0.306	0.181				
Immunoglobulin kappa joining 1	-0.0978	0.0748	0.202				
OGTT blood glucose 30 minutes	-0.174	0.056	0.00424				
Total cholesterol	0.226	0.0839	0.0118				
C-reactive protein	-0.0331	0.0143	0.0278				
Alanine aminotransferase	0.0139	0.00617	0.0323				
Aspartate aminotransferase	-0.0197	0.0096	0.0499				
Fasting blood glucose	-0.272	0.136	0.0550				
Insulin	-0.0400	0.0237	0.103				
C-peptide	0.000739	0.000493	0.145				

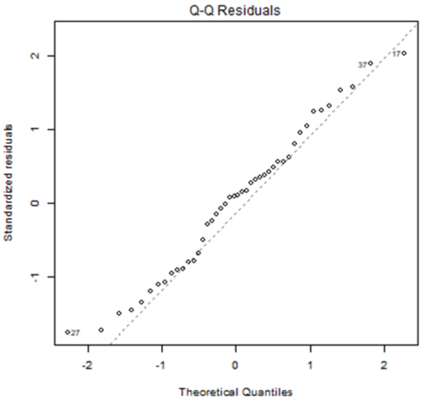

Supp Table 4
